# Structural and Functional Characterization of the Recombinant Death Domain from Death-Associated Protein Kinase

**DOI:** 10.1371/journal.pone.0070095

**Published:** 2013-07-29

**Authors:** Evangelos Dioletis, Andrew J. Dingley, Paul C. Driscoll

**Affiliations:** 1 Research Department of Structural & Molecular Biology**,** University College London, London, United Kingdom; 2 School of Chemical Sciences and School of Biological Science, The University of Auckland, Auckland, New Zealand; 3 ICS-6 (Structural biochemistry), Research Center Jülich, Jülich, Germany; 4 Division of Molecular Structure, Medical Research Council National Institute for Medical Research, London, United Kingdom; University of Oulu, Finland

## Abstract

Death-associated protein kinase (DAPk) is a calcium/calmodulin-regulated Ser/Thr-protein kinase that functions at an important point of integration for cell death signaling pathways. DAPk has a structurally unique multi-domain architecture, including a C-terminally positioned death domain (DD) that is a positive regulator of DAPk activity. In this study, recombinant DAPk-DD was observed to aggregate readily and could not be prepared in sufficient yield for structural analysis. However, DAPk-DD could be obtained as a soluble protein in the form of a translational fusion protein with the B1 domain of streptococcal protein G. In contrast to other DDs that adopt the canonical six amphipathic α-helices arranged in a compact fold, the DAPk-DD was found to possess surprisingly low regular secondary structure content and an absence of a stable globular fold, as determined by circular dichroism (CD), NMR spectroscopy and a temperature-dependent fluorescence assay. Furthermore, we measured the *in vitro* interaction between extracellular-regulated kinase-2 (ERK2) and various recombinant DAPk-DD constructs. Despite the low level of structural order, the recombinant DAPk-DD retained the ability to interact with ERK2 in a 1∶1 ratio with a *K*_d_ in the low micromolar range. Only the full-length DAPk-DD could bind ERK2, indicating that the apparent ‘D-motif’ located in the putative sixth helix of DAPk-DD is not sufficient for ERK2 recognition. CD analysis revealed that binding of DAPk-DD to ERK2 is not accompanied by a significant change in secondary structure. Taken together our data argue that the DAPk-DD, when expressed in isolation, does not adopt a classical DD fold, yet in this state retains the capacity to interact with at least one of its binding partners. The lack of a stable globular structure for the DAPk-DD may reflect either that its folding would be supported by interactions absent in our experimental set-up, or a limitation in the structural bioinformatics assignment of the three-dimensional structure.

## Introduction

The expression of the gene encoding death-associated protein kinase (DAPk; HUGO Gene Nomenclature Committee identifier: DAPK1) was originally identified as being essential for the execution of apoptosis induced by IFN−γ [1]. Research has shown that DAPk acts as a positive mediator of cell death induced by a range of stimuli including tumor necrosis factor-α (TNF-α), anti-CD95/Fas agonists, transforming growth factor-β (TGF-β), the dependence receptor UNC5H2 in the absence of netrin-1, short-chain acyl ceramide derivatives, endoplasmic reticulum-stress [2], detachment from the extracellular matrix and oncogene-induced hyperproliferative signals [3], [4], [5], [6], [7], [8], [9], [10]. Together these observations suggest that DAPk functions at an important point of integration for different cell death signaling pathways. DAPk is a 160 kDa calcium/calmodulin-regulated Ser/Thr-protein kinase with unique domain architecture. DAPk consists of an N-terminal catalytic domain, a calmodulin (CaM)-binding segment, eight ankyrin repeats, a GTP-binding Ras of complex proteins (Roc) domain, a C-terminal of Roc (COR) domain, a death domain (DD), and a serine-rich (SR) C-terminal tail [1], [3], [4], [11],[12]. A variety of evidence indicates that the DD-region of DAPk (DAPk-DD) exerts a regulatory role on the apoptotic function of DAPk. Widespread apoptosis is observed when human embryonic kidney (HEK) 293 cells are transiently transfected with a constitutively active DAPk mutant that lacks the calmodulin regulatory element (DAPk/ΔCaM) [3], [4]. In contrast, apoptosis is significantly reduced when HEK 293 cells or MCF7 human breast carcinoma cells are transfected with a DAPk/ΔCaM mutant lacking the DD [4]. Additionally, cell death was attenuated in HEK 293 cells co-transfected with the expression constructs DAPk/ΔCaM and DAPk-DD (comprising just the death domain residues), suggesting that the over-expressed DAPk-DD can act in a dominant negative fashion perhaps by sequestering factors required for endogenous DAPk activity [4].

The death fold superfamily of proteins includes the following four families: death domains (DDs), death effector domains (DEDs), caspase recruitment domains (CARDs) and PYRIN domains (PYDs). Whilst these protein families can be distinguished on the basis of pairwise sequence similarities, structure determination of representative members of each family has revealed that they share a common architecture consisting of six amphipathic α-helices (H1−H6) arranged in a compact, mostly antiparallel topology in which the N- and C-terminii are close together. Within each of the death fold families, the buried residues of H2 through H5 exhibit relatively high sequence similarity, whereas H1 and H6 are more highly divergent. The primary function of the death fold superfamily of protein modules is to mediate protein-protein interaction by forming predominantly homotypic associations, thereby underpinning the formation of multi-subunit signaling complexes. The extensive amino acid sequence diversity of surface-exposed residues in the death folds provides a wide range of physicochemical properties. Nevertheless most if not all contacts observed in DD complexes appear to fall into one of three topologically similar arrangements denoted: (i) type I, negatively charged residues of H2 and H3 of one DD interact with positively charged residues of H1 and H4 of another DD; (ii) type II, H4 and the H4–H5 loop of one DD interact with the N-terminal end of H6 of another DD; and (iii) type III, H3 of one DD interacts with the H1–H2 and H3–H4 loops of a second DD [13]. Prominent examples of the interfacial interactions between DDs are provided by the PIDDosome core complex composed of the DDs of PIDD and RAIDD [14], the highly related core of the CD95/Fas death-inducing signaling complex (DISC) comprising CD95-DD and FADD-DD [15], [16], [17], and the Myddosome comprising the DDs of MyD88, IRAK2 and IRAK4 [18].

**Figure 1:**
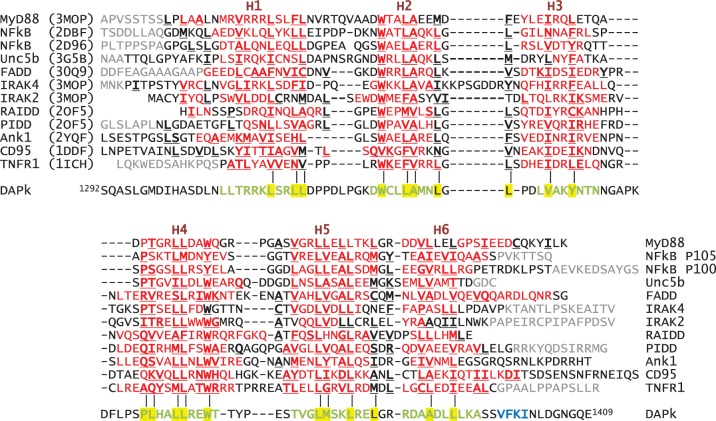
Sequence alignment DAPk-DD against death domains with known 3D structures. Residues assigned in helical secondary structures H1 through H6 (dark red labels) are shown in red, and those with buried side chains are shown in bold and underlined. Residues present in the native protein but not included or visible in the corresponding structure are shown in grey. The alignment has been manually curated on the basis of a multiple superposition of the 3D coordinates. The sequences are displayed in rank order of sequence identity to DAPk-DD: (top) MyD88 (PDB code 3MOP); NF-κB p105 subunit (2DBF); NF-κB p100 subunit (2D96); Unc5b (3G5B); FADD (30Q9); IRAK4 (3MOP); IRAK2 (3MOP); RAIDD (2OF5); PIDD (2OF5); ankyrin1 (2YQF); CD95/Fas (1DDF); TNF receptor-1 (1ICH). The DAPk-DD region is shown at the bottom; the result of sequence-based secondary structure prediction (JPRED) is indicated as bold green letters for α-helix and bold blue letters for β-strand; yellow backgrounding highlights unambiguous conservation of hydrophobic buried residues compared to the other DD sequences. Note the difficulty to assign such conservation for the putative H6 of DAPk-DD, as well as a uniquely long H3-H4 loop.

Secondary structure prediction and sequence analysis for the DAPk-DD region suggests the presence of the expected six α-helices. Moreover hydrophobic residues are appropriately placed to align with key residues whose side chains are known to constitute the core of multiple examples of DD 3D structures (Figure 1). Particularly notable is the presence of the two tryptophan residues (Trp1329 and Trp1367), towards the N-terminus of H2 and the C-terminus of H4 respectively, that are strongly conserved amongst other DDs. The DAPk-DD has been reported to interact with the DD-containing intracellular portion of the netrin-1 receptor UNC5H2 *in vivo* [7], the extracellular signal-regulated kinases-1 and -2 (ERK1/2) *in vivo* and *in vitro* [19], and the MAP/microtubule affinity-regulating kinases-1 and -2 (MARK1/2) [20]. Further, DAPk-DD has been suggested to be important for the interaction of DAPk with tuberous sclerosis-2 (TSC2) [21] and pyruvate kinase M2 (PKM2) [22].

In order to further elucidate its role in the regulation of the DAPk function we set out to investigate the three-dimensional structure of the DAPk-DD. Recombinant DAPk-DD was over-expressed as a soluble fusion protein in *E. coli*, purified and subjected to analytical gel filtration, circular dichroism (CD), NMR spectroscopy and a temperature-dependent fluorescence assay. The DAPk-DD was found to be a monomer at low micromolar concentrations that has surprisingly low regular secondary structure content, which has not been seen previously for other members of the DD family. Furthermore, we probed the interaction between ERK2 and various recombinant DAPk-DD constructs through *in vitro* binding and isothermal titration calorimetry (ITC) experiments. Despite the observed low level of structural order, the recombinant DAPk-DD retains the ability to interact with ERK2 in a 1∶1 ratio with a *K*_d_ in the low micromolar range. Taken together our data argue that the DAPk-DD, when expressed in isolation, is not stable as a classical 3D DD fold, yet in this state retains the capacity to interact with at minimum one of its reported binding partners.

## Materials and Methods

### Generation of the Expression Constructs

DAPk-DD expression constructs were prepared from the full-length human DAPk cDNA inserted into the pcDNA3 vector, kindly provided by Prof. A. Kimchi (Weizmann Institute, Israel). The DAPk-DD DNA sequence (residues 1293−1399) was amplified using the forward (5′-GATGATGAT GGATCCTCA CAGGCCAGC CTCGG-3′) and reverse (5′-ATCATCATC CTCGAGCAC AGAGGATGC CTTCAGC-3′) primers, and inserted between the *BamH*I and *Xho*I sites in the plasmid GEV2, which encodes an N-terminal appendage of the B1 immunoglobulin binding domain of streptococcal protein G (GB1), a well-known solubilization enhancement tag [23]. The construct also encodes a thrombin target sequence between the GB1 and DAPk-DD domains and a C-terminal His_6_-tag. The corresponding expressed protein product is denoted GB1-DAPk-DD(S).

A second, longer DAPK-DD expression construct, GB1-DAPk-DD(L), comprising residues 1293−1408 was prepared by mutagenesis of a separately prepared GEV expression vector that includes the DAPk-DD and the whole Ser-rich tail (residues 1293−1431) using the 5′-primer 5′-ATCATCATC CTCGAGCCG GGATACAAC AGAGC-3′. Mutagenesis was performed using the QuikChange site-directed mutagenesis kit (Stratagene, La Jolla, CA, USA) with forward (5′-GTGTTCAAA ATCAACCTG GATGGCAAT GGCCTCGAG CACCACCAC CACCACCAC TGAGATCC-3′) and reverse 5′-GGATCTCAG TGGTGGTGGT GGTGGTGCT CGAGGCCAT TGCCATCCA GGTTGATTT TGAACAC-3′) primers. Preparation of the truncated GB1-DAPk-DD constructs used for ERK-2 binding experiments was carried out in a similar fashion. All of these constructs also included a C-terminal His_6_-tag and a thrombin recognition sequence between the GB1 and DAPk-DD domains.

The template DNA used for the generation of the FADD-DD (residues 93−192) PCR insert was described previously [24]. Two rounds of PCR were performed to introduce a tobacco etch virus (TEV) cleavage site at the N-terminus of FADD-DD: first round PCR, 5′-CCTGTATTT TCAGGGCGG GGAAGAAGA CCTG-3′ (forward) and 5′-ATCATCATC GAATTCTCA ACTCCTGTT CTGGAGG-3′ (reverse); second round PCR forward primer 5′-AATGATGAT GGATCCGAA AACCTGTAT TTTCAGGGC-3′. The final PCR product was ligated (*BamH*I and *EcoR*I) into pET-28GB1-1 (pET28a incorporating the GB1 coding sequence inserted between *Nde*I and *BamH*I sites). The resulting protein product is His_6_-(thrombin site)-GB1-(TEV site)-FADD-DD. The GB1-CD95-DD-His_6_ protein used in thermal denaturation experiments was kindly provided by Dr Diego Esposito (MRC National Institute of Medical Research, London).

The expression vector for full length human ERK2, encoded in the pGEX-6-P3 vector (GE Healthcare Life Sciences) as an N-terminal glutathione-S-transferase (GST)-tagged construct with a PreScission protease site, was a kind gift from Prof. Dario Alessi (The MRC Protein Phosphorylation Unit, Dundee).

### Protein Expression and Purification

All expression vectors were transformed into *E. coli* BL21 codon plus cells (Stratagene). GB1-DAPk-DD proteins were expressed in the soluble phase and purified by Ni-IMAC and size-exclusion chromatography (Figure S1). ^15^N-Isotope labeling was accomplished using M9 medium containing ^15^N-ammonium salts supplemented with trace levels of metal ions and vitamins. The expression and purification of the truncated GB1-DAPk-DD constructs were performed using the same protocol.

The expression and purification of the GB1-FADD-DD construct was performed as described above with minor modifications. FADD-DD was obtained by treating purified GB1-(TEV site)-FADD-DD with His_6_-tagged TEV protease (Invitrogen), followed by separation of the cleaved GB1 and TEV protease by passing the mixture through a Ni-NTA column.

Large scale production of recombinant ERK2 was carried out as previously described [25]. The molecular mass of the protein samples was verified by electrospray-ionization mass spectrometry. The concentration of the purified proteins was determined by measuring the UV absorbance at 280 nm. The theoretical extinction co-efficient for each protein was calculated using the ExPASy ProtParam tool [26].

### Analytical Size Exclusion Chromatography

Analytical size exclusion chromatography (SEC) experiments were performed using a pre-packed Superose 12 HR 10/30 column (∼24 ml bed volume) connected to an AKTA Purifier HPLC system (GE Healthcare Life Sciences) pre-equilibrated with the protein purification buffer. Elution profiles were recorded by monitoring the absorbance at 280 nm and elution volumes determined using the Unicorn software. Samples were centrifuged at 17,900 *g* for 10 min prior to injection to remove insoluble material. One-hundred microliter aliquots (0.1 to 20 mg ml^−1^) were sampled twice for each protein concentration. The flow rate was 1 ml.min^−1^ and experiments were run at room temperature. The column was calibrated using gel filtration molecular weight standards (Bio-Rad Laboratories). The protein standards were reconstituted in the same buffer used to equilibrate the column, but without dithiothreitol (DTT) to avoid disruption of disulfide bonds.

### Circular Dichroism Spectropolarimetry

Circular dichroic (CD) spectra were acquired using a stopped-flow circular dichroism spectropolarimeter model 202SF (Aviv Instruments Inc.), with a CFT-23 recirculating chiller cooling unit (Neslab Instruments Inc.). Aviv CD v2.76 software was used to calculate the molar ellipticity. Spectra were recorded over the wavelength range of 190−260 nm. The path-length of the quartz cuvette was 0.1 mm. The bandwidth was 1 nm, settling time 0.3 sec, the averaging time 3 sec, and the wavelength step 0.5 nm. All samples were dialyzed overnight at 4°C. The protein sample concentration ranged from 10 to 50 µM and the CD spectra were normalized to yield mean residue ellipticity. All spectra were recorded at 25°C, unless otherwise stated. Samples were pre-equilibrated for 15 min prior to running the experiments. For each measurement three scans were averaged and subtracted from a ‘blank’ obtained for the buffer alone. The error bars plotted in the CD profiles represent an estimate of the uncertainty of the measurements derived from the three scans.

Thermal denaturation CD profiles were acquired by monitoring the ellipticity at 222 nm as a function of temperature employing a constant scan rate of 1 deg.min^−1^ over the range 5–95°C. The reversibility of the thermal unfolding process was evaluated by recording the far UV spectrum upon cooling the protein to 5°C. The dynode voltage was monitored continuously to determine the useful isothermal wavelength range for spectral measurements and to evaluate the integrity of the protein samples during thermal denaturation. Where cooperative thermal unfolding was observed as a two-state process, the respective melting temperature (*T*_m_) and free energy change (Δ*G*_U→F_) were derived by van’t Hoff analysis of the denaturation profiles.

### NMR Spectroscopy

NMR experiments were recorded on Varian Inova spectrometers (Agilent Technologies) operating at 600 and 800 MHz proton frequencies. The spectrometer was equipped with either a 5-mm z-gradient ^1^H/^15^N/^13^C standard room temperature probe (600 MHz), or a HCN cold probe (800 MHz). Spectra were recorded at 25°C. Protein samples were prepared in 20 mM NaPi, 50 mM NaCl, 1 mM DTT, 1 mM EDTA, 7% D_2_O, pH 6.5. The protein concentration was 46 µM.

Two-dimensional (2D) ^1^H**−** ^15^N heteronuclear single quantum coherence (HSQC) spectra were recorded using a pulse-field gradient sensitivity-enhanced pulse scheme with the incorporation of water flip-back pulses. The data matrix consisted of 128*×384* data points (were n* refers to complex points) with acquisition times of 61 ms (*t*_N_) and 96 ms (*t*_HN_). A total of 32−128 scans per complex *t*_N_ increment was collected. The recycle delay was 1.2 s. The total measuring time was between 1.1 and 4.5 h.

All raw NMR data were processed using nmrPipe [27] and visualized in nmrDraw. Peak picking and data analysis were performed using the Analysis software program within the CCPN suite [28].

### Thermal Denaturation Monitored by Fluorescence

Thermal denaturation experiments were recorded on an iQ5 Real-Time PCR Detection System controlled by the iQ5 optical system software version 1.0 (Bio-Rad Laboratories). Twenty-five microliters of a Sypro Orange 5×stock solution (Molecular Probes) were mixed with 25 µl of each protein sample to a final protein concentration of 50 µM. The fluorophore-mixed protein samples were loaded into an iQ5 96-well plate. The plate was heated from 25 to 85°C in increments of 0.5°C with a dwell time of 10 sec. Three samples for each different protein concentration were simultaneously assessed. The buffer conditions for GB1-DAPk-DD(L) were: 10 mM NaPi (pH 7.4), 150 mM NaCl, 0.5 mM DTT and 0.5 mM EDTA. The final buffer conditions for GB1-CD95-DD were: 5 mM Tris (pH 7.0), 25 mM NaCl, 0.5 mM EDTA and 1 mM β- mercaptoethanol.

### GST Pull-down Assay

Fifty microliters (settled volume) of the GST beads (Novagen) were aliquoted into separate microcentrifuge tubes, centrifuged at 1,960 *g* for 6 min and equilibrated with 1 ml of the assay buffer. The assay buffer used was 50 mM HEPES (pH 7.5), 50 mM NaCl, 1% IGEPAL CA630, 20% glycerol, 10 mM EDTA and 1 mM DTT. Proteins used in the in vitro pull-down assays were derived from 5 ml LB cultures (37°C, 250 rpm). Cell cultures were centrifuged and the cell pellets resuspended in 1 ml of the assay buffer. The cells were lysed by sonication and the lysates centrifuged at 17,900 *g* for 10 min. The cell supernatants were applied to the GST beads and incubated at 4°C for 2 h. Following centrifugation, cell lysates were decanted and the beads were washed seven times with 1 ml of the assay buffer. Fifty microliters of the elution buffer (50 mM Tris pH 8.0, 10 mM reduced glutathione) were incubated with the beads for 10 min, centrifuged and the supernatant stored separately. The elution step was repeated twice and the three eluates were pooled.

### Western Blotting

The His-tag monoclonal antibody (Novagen) and the accompanying colorimetric detection protocol were used for the Western blots. Incubation with the His-tag antibody was for 1 h and the blocking solution contained 5% milk. Seeblue2 protein markers (Invitrogen) were used for SDS-PAGE separation.

### Isothermal Titration Calorimetry

For isothermal titration calorimetry (ITC) measurements the protein samples were extensively dialyzed against 50 mM HEPES (pH 7.5), 50 mM NaCl, 1 mM TCEP and 1 mM EDTA, and concentrated to the required level. Experiments were performed with a VP-ITC calorimeter (MicroCal): 19 aliquots of 15 µl of 159 µM GB1-DAPk-DD(L), or 250 µM of GB1-DAPk-DD(S), or 100 µM GB1 were injected into 1.45 ml of 8.85−18.6 µM ERK2 (different concentrations of ERK2 were employed for each titration) at 25°C. Experiments were repeated twice. Following subtraction of the corresponding heats of dilution, the binding isotherms were fitted using nonlinear least-squares optimization to obtain three parameters: binding stoichiometry (*n*), enthalpy of interaction (Δ*H*), and the equilibrium dissociation constant (*K*_d_). Data processing was performed with the Origin ITC analysis software package supplied by MicroCal.

### Bioinformatic Analysis

Structure-based sequence alignments were performed with the online program DALI Lite [29] using p75ICD-DD and the FADD-DD as templates. Collation of sequence alignments was performed with BioEdit. The DAPk-DD sequence was aligned to that of p75ICD-DD using the program ClustalW [30].

## Results

### Design and Expression of the DAPk-DD Construct

A limited screening of the DD boundaries was performed. Boundaries tested were based on bioinformatics analysis of DD sequences and available structural data of DDs. The boundaries of the constructs prepared were derived based on Pfam, SMART and simple sequence alignments with other death domains. Pfam, in particular, predicted that the DAPk-DD to span residues 1312−1397 (E-value = 2.7×10^−20^), whereas SMART estimated the DAPk-DD to be longer at the amino terminus beginning at position 1300 and ending at position 1397 (E-value = 5.90×10^−25^). In addition, a structure-based sequence alignment was performed and found to be in agreement with the sequence alignment. In this report, two DAPk-DD constructs are presented: DAPk-DD(S) (residues 1293−1399) and DAPk-DD(L) (1293−1408). Both constructs cover the putative DD boundaries defined by the bioinformatics analysis, therefore ensuring the inclusion of the complete core. Multiple strategies to express DAPk-DD as an isolated domain using overexpression in an *E. coli* system produced insoluble protein products. Attempts to refold the inclusion body material yielded negligible quantities of soluble product. To enhance the solubility we expressed DAPk-DD as a translational fusion protein at the C-terminus of the bacterial protein GB1, a well-characterized ‘solubility-enhancing tag’ that has been demonstrated to assist the recovery of soluble protein for a range of different proteins in *E. coli* [31], including the wild-type DD of CD95/Fas and the PYD of NALP1 [23], [32]. Two different fusion constructs were generated, GB1-DAPk-DD(S) and GB1-DAPk-DD(L). The designations (S) and (L) refer to short and long variants of the DAPk-DD sequence respectively (Figures 1, see Methods). GB1-DAPk-DD(S) includes all the residues predicted to form α-helical structure, whereas GB1-DAPk-DD(L) has a nine-residue C-terminal extension derived from the Ser-rich tail. The inclusion of the complete Ser-rich region led to vanishingly small quantities of expressed protein. All of the experiments presented here were conducted with intact GB1-DAPk-DD fusion constructs; proteolytic removal of GB1 using thrombin resulted in the precipitation of the DAPk-DD portion. The GB1-DAPk-DD constructs were purified away from the cellular milieu by Ni-IMAC and size-exclusion chromatography (SEC). Figure S1 shows the results of a size-exclusion chromatogram and the collected fractions run on an SDS-PAGE. The results show that the GB1-DAPk-DD protein can be purified to near homogeneity using this protocol. The pooled fractions were further analyzed by analytical SEC at 1 mg/ml and found to elute at a similar elution volume to GB1-FADD-DD (Figure S1C), which is known to not self-associate (see below).

### DAPk-DD Reversibly Oligomerizes in vitro

**Figure 2:**
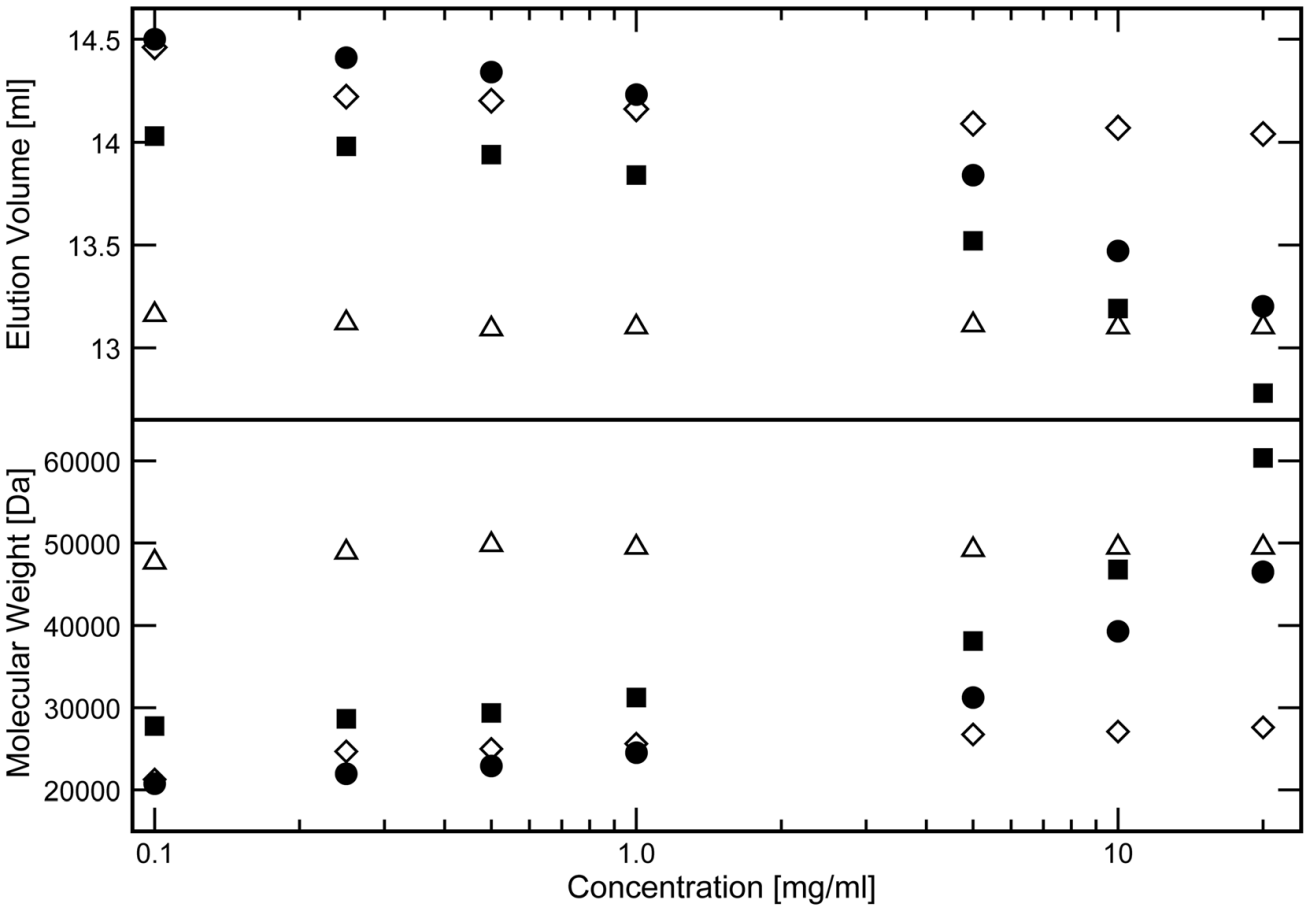
The DAPk-DD shows concentration dependent oligomerization. (A) Control proteins GB1-FADD-DD (open diamonds) and ovalbumin (open triangles) along with GB1-DAPk-DD(S) (filled circles) and GB1-DAPk-DD(L) (filled squares) constructs were run at seven different concentrations through a Superpose-12 analytical gel filtration column and the elution volumes were plotted. (B) The elution volumes shown in (A) were converted into apparent MWs using the calibration curve (Figure S3). Two sets of data were collected for each sample and the average value shown. Elution volume differences between sets were ≤0.04 ml.

The *in vitro* oligomerization state of the expressed GB1-DAPk-DD proteins was examined by analytical SEC. The elution profiles of the two GB1-DAPk-DD constructs as well as two control proteins, a homologous GB1-FADD-DD construct and ovalbumin, were measured as a function of protein concentration (Figure 2A, Figure S2). The elution volume of the two GB1-DAPk-DD constructs decreased as the protein concentration increased, contrasting with the results for ovalbumin and GB1-FADD-DD that showed essentially invariant elution volumes over the concentration range examined. For the DAPk-DD proteins a minimum elution volume was not reached suggesting that these proteins possess the potential for high order self-association. The tailing of the elution peak profile to earlier elution volumes for the DAPk-DD constructs (Figure S2) is indicative of the presence of a mixture of oligomerization states; the population of the higher order species appears to increase as the protein concentration increases. Conversion of the elution volumes into apparent molecular weights (Figure 2B) shows that the GB1-DAPk-DD(S) protein varies between effective masses of ∼21 and ∼47 kDa (predicted monomer molecular mass 19.6 kDa), and the GB1-DAPk-DD(L) varies between ∼27 and 61 kDa (predicted monomer molecular mass 20.6 kDa). These results suggests that at low concentrations the two GB1-DAPk-DD constructs adopt hydrodynamic dimensions resembling those of a monomer similar to the homologous GB1-FADD-DD (molecular mass estimated from SEC is 21.3 kDa; calculated monomer mass 20.9 kDa), whereas at higher concentrations they approach (and even exceed) the hydrodynamic behavior of ovalbumin (molecular mass estimated from SEC is 46.5 kDa; calculated molecular mass 44 kDa).

Since the protein samples were initially concentrated to 20 mg ml^−1^ and subsequently diluted for the SEC measurements, the apparent oligomerization of GB1-DAPk-DD proteins must be reversible. Consequently, GB1-DAPk-DD appears to be able to form oligomers that depend on the sample concentration and therefore in a dynamic equilibrium between monomeric and higher order species. The GB1 domain itself is well known to be stably monomeric and the elution volume of the GB1-FADD-DD control construct shows minor variation with concentration. A general search in the literature and query of the on-line Biomolecular Interaction Network Database (BIND) [33], which archives all known biomolecular interactions and complexes that arise from published experimental research, did not return any reported interactions between GB1 and other proteins. Moreover characterization of the NMR spectrum of the GB1-DAPk-DD proteins (see below) shows that the GB1 component is unperturbed relative to the isolated GB1 domain. GB1 self-association cannot be responsible for the observed aggregation of GB1-DAPK-DD at higher concentrations, Hence, self-association of the GB1-DAPk-DD proteins is not due the GB1 domain itself, or between the GB1 domain and the appended DAPk-DD polypeptide either in *cis* or in *trans*, and therefore must be a property of the DAPk-DD sequence itself. This finding is broadly consistent with the observation that DAPk-DD polypeptides, when expressed in isolation, spontaneously aggregate in inclusion bodies. Furthermore we can safely attribute the properties of the GB1-DAPk-DD fusion proteins in further biophysical analyses as arising from the sum of the (GB1 and DAPk-DD) parts.

### DAPk-DD Adopts an Intrinsically Disordered State with Partial Helical Structure

**Figure 3:**
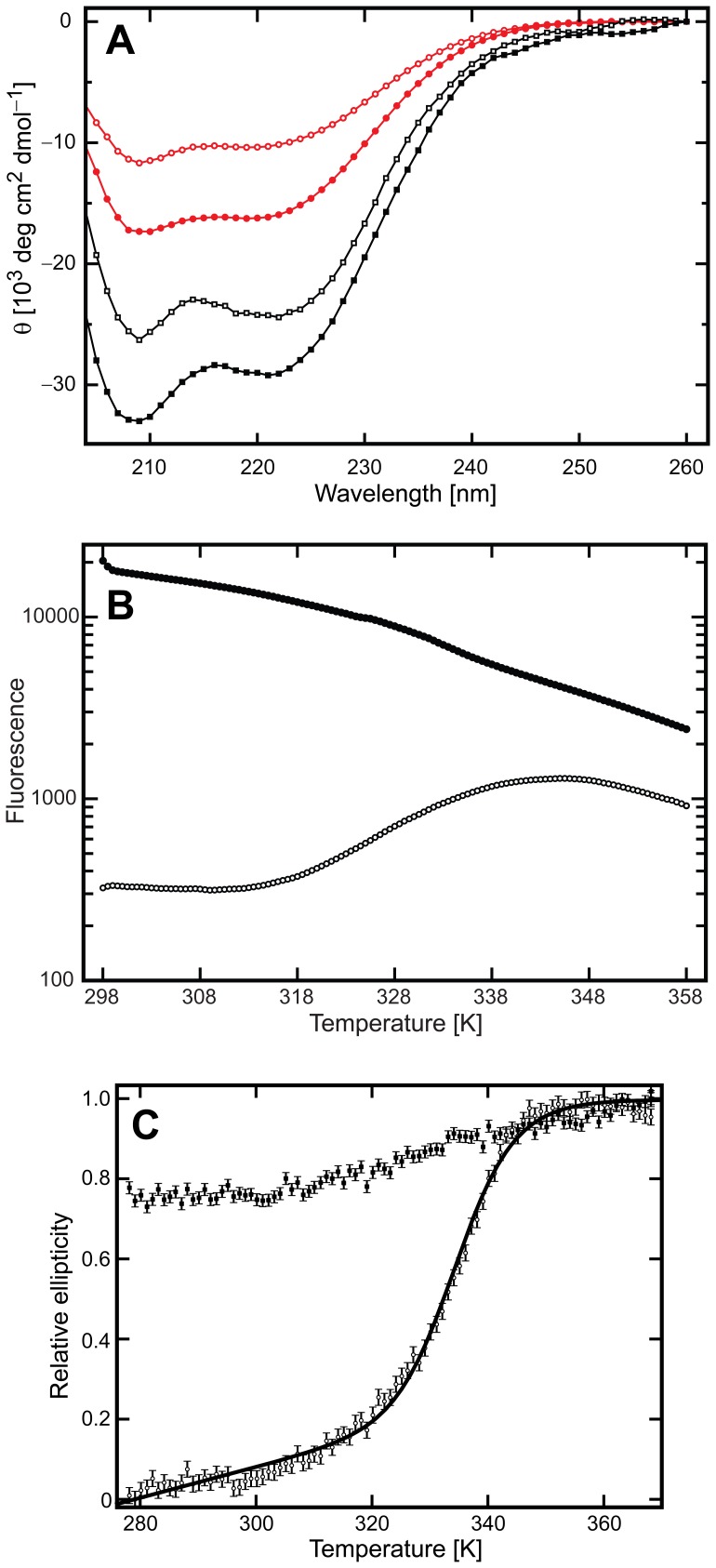
The DAPk-DD has intrinsically disorder protein properties. (A) Far-UV CD data of the GB1-corrected native DAPk-DD(L) (blue circles) and in the presence of 40% v/v TFE (green filled circles). The addition of 40% v/v TFE increased the DAPk-DD helicity by ∼56% based on the 222 nm reading. The spectra for the GB1-subtracted FADD-DD in the absence (red circles) and presence of TFE (black filled circles), and an increase of ∼18% in helicity was observed. Data collection in the presence of TFE were terminated at 204 nm because of the high tension voltage (dynode) registered at lower wavelengths. The high tension voltage arose from the buffer components, i.e., 20 mM sodium phosphate buffer, 150 mM NaCl, and 1 mM DTT, pH 7.4. Error bars are estimates of uncertainties derived from the three scans recorded per sample. (B) The fluorimetric thermal denaturation profile of the GB1-DAPk-DD(L) protein (solid circles) resembles a protein that is loosely structured with the fluorophore able to readily interact with hydrophobic residues. As a control of a DD with the canonical six-helix bundle fold, the fluorimetric thermal denaturation profile of the GB1-CD95-DD protein is shown (open circles). (C) Far-UV CD thermal denaturation of GB1-DAPk-DD(L) (filled circles) and GB1-FADD-DD (open circles). The thermal denaturation profile of the GB1-corrected DAPk-DD features a long transition indicating that the protein unfolds in a noncooperative manner, whereas the GB1-corrected FADD-DD shows a denaturation profile indicative of cooperative unfolding. The line represents a fit of the FADD-DD data by a van’t Hoff analysis, with derived *T*_m_, Δ*H* and Δ*S* values of 62±0.3°C, −182.7±6.5 kJ mol^−1^ and 0.55±0.02 kJ K^−1^ mol^−1^, respectively.

**Figure 4:**
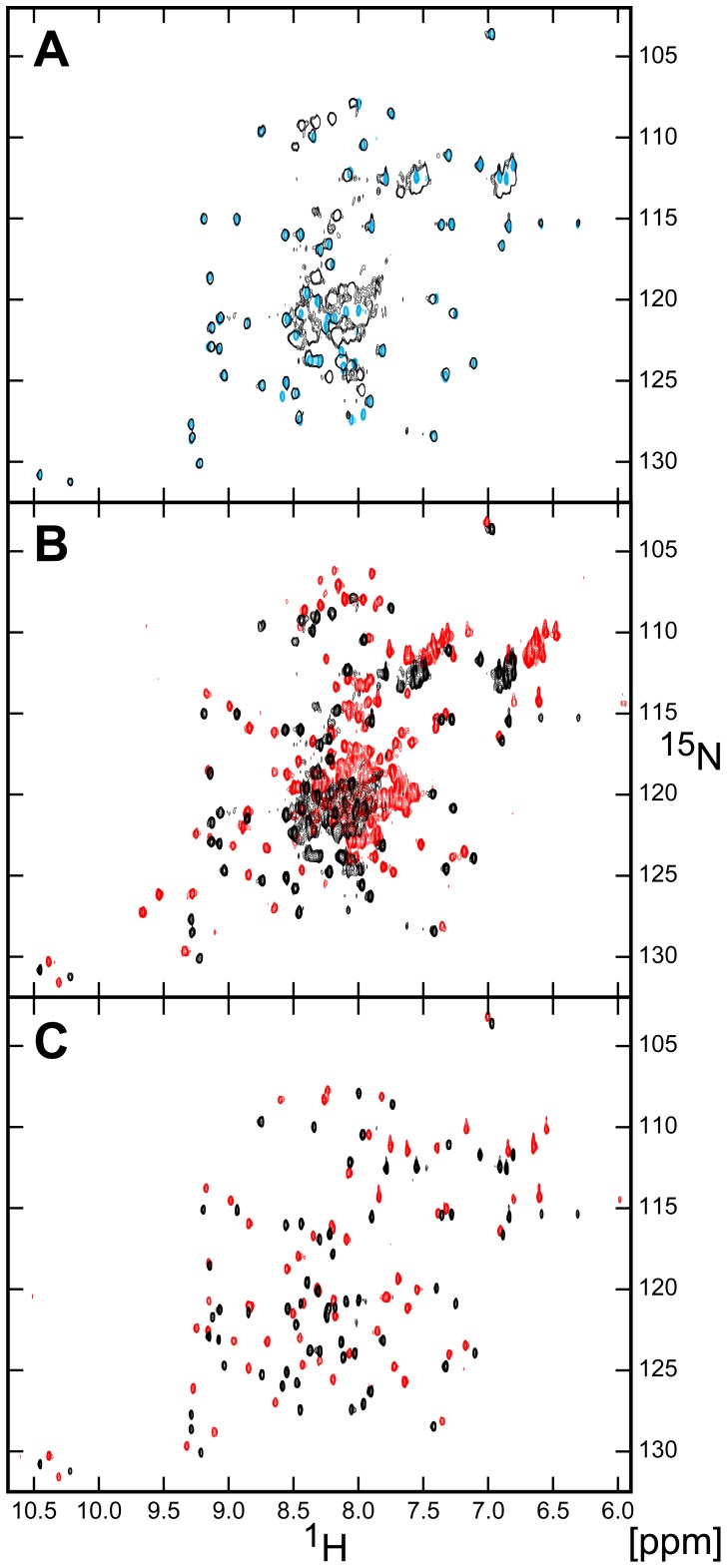
Overlay of the 2D ^1^H-^15^N HSQC spectra of ^15^N-labeled GB1-DAPk-DD and ^15^N-labeled GB1 in the presence and absence of TFE. (A) Overlay of ^15^N-labeled GB1-DAPk-DD (black) and ^15^N-labeled GB1 (cyan) spectra shows that the chemical shifts of the resonances arising from the GB1 residues in both constructs have near identical chemical shifts, therefore indicating that there are negligible contacts between GB1 and the DAPk-DD in the GB1-DAPk-DD construct. (B) Overlay of HSQC spectra of ^15^N-labeled GB1-DAPk-DD in the absence (black) and presence (red) of 40% (v/v) TFE. The majority of the dispersed, intense signals arise from GB1. The unresolved resonances in the center of the spectrum arise from the DD and indicate that the domain lacks a tertiary fold. The addition of TFE leads to a general decrease in resonance line widths and a minor increase in resonance dispersion, suggesting that TFE aids the formation of secondary structure elements of the DD, but the domain lacks a compact DD fold. (C) Overlay of HSQC spectra of ^15^N-labeled GB1 in the absence (black) and presence (red) of 40% TFE (v/v).

The CD spectrum of GB1-DAPk-DD(L) was recorded and corrected for the contribution from the GB1 domain (Figure 3A). The overall characteristic of this spectrum is typical of a protein with a significant contribution of α-helical secondary structure, with a (negative) peak at ∼208 nm and a shoulder at 222 nm. We separately recorded the 2D ^15^N-^1^H HSQC NMR spectrum of isotope-labeled GB1-DAPk-DD(L) (Figure 4A,B). The spectrum contains a subset of well-dispersed cross peaks supplanted by a larger number of essentially unresolved resonances at ^1^H chemical shift of ∼8.0–8.4 ppm (Figure 4A,B). Analysis of this spectrum and comparison with the spectrum of the isolated GB1 domain (Figure 4A,C) and other well-characterized GB1-fusion proteins (not shown) indicates that the resolved cross peaks primarily correspond to the GB1 portion of the fusion protein. The poorly resolved subset of resonances that derive from the DAPk-DD portion of the polypeptide indicate a lack of ordered globular structure. For comparison we recorded the GB1-corrected CD spectrum of GB1-FADD-DD (Figure 3A); the spectrum has a similar profile of that for GB1-DAPk-DD(L), but possesses a significantly greater (negative) amplitude: at 222 nm, the GB1-corrected FADD-DD spectrum has a mean residue ellipticity (MRE) of −24,700 deg.cm^2^.dmol^−1^; (MRE = −19,200 deg.cm^2^.dmol^−1^ for a FADD-DD-only construct, data not presented), whereas the corresponding value for DAPk-DD(L) is −10,250 deg.cm^2^.dmol^−1^. CD spectroscopy measurements have been previously reported for both FADD-DD and CD95-DD [34], with MRE values at 222 nm of ∼−18,000 deg.cm^2^.dmol^−1^ respectively, in reasonable agreement to the values obtained here for FADD-DD. Similarly, the death domain of the *Drosophila* protein Pelle was reported to possess a CD spectrum showing double minima at 209 and 223 nm, with a MRE (223 nm) of ∼−18,000 deg cm^2^ dmol^−1^ [35]. The observation that the absolute ellipticity for the DAPk-DD(L) is more than 50% lower than observed for the FADD-DD indicates that the DAPk-DD does not exhibit the same level of α-helical secondary structure. Similar results were obtained for the shorter GB1-DAPk-DD(S) construct (data not shown). The CDSSTR program [36], [37], [38], [39] was used to estimate the helical content of the proteins by deconvolution of the CD spectra. CDSSTR predicted 72% α-helical content for FADD-DD, a value that closely matches the helical content observed for the 3D solution structure of the FADD-DD [24]. In contrast, CDSSTR predicts only 39% α-helical content for the DAPk-DD(L) protein.

To further assess the conformational status of the DAPk-DD we obtained the fluorimetric thermal denaturation profiles using the ‘Thermofluor’ method [40], [41]. Control experiments using GB1-CD95-DD exhibited a characteristic sigmoid transition corresponding to thermal denaturation of the CD95-DD, which adopts a canonical 3D DD structure, with a midpoint of ∼332 K (Figure 3B). In contrast, the data for GB1-DAPk-DD(L) indicated that the fluorescence signal was high even at the starting (low) temperature, and failed to yield an observable unfolding transition as the sample temperature was raised. The profile is typical of a protein that is poorly ordered and lacks a stable tertiary fold; the fluorophore readily binds the disordered protein even at a low temperature (Figure 3B). We similarly measured the thermal denaturation profiles of GB1-DAPk-DD(L) and GB1-FADD-DD by CD spectroscopy (Figure 3C). Full CD spectra of GB1-DAPk-DD(L) were also recorded at 5, 25 and 95°C to check that the CD profile did not show any major change due to a shift in secondary structure type (Figure S4). The spectral profiles do not change noticeably over this temperature range, except for the reduction in CD ellipticity values due to a loss of secondary structure caused by thermal denaturation. At each measurement point the data were corrected for the contribution from the isolated GB1 domain (for which the data were recorded separately). As expected, the profile obtained for FADD-DD displays a sigmoidal transition consistent with loss of helical structure and highly cooperative unfolding of the death domain (Figure 3C). The thermal mid-point *T*_m_ and Δ*G*_U→F_ terms for the FADD-DD derived from a van’t Hoff analysis of the data are 335 K and −19.82 kJ mol^−1^respectively. The thermal denaturation profile of the GB1-corrected DAPk-DD profile features a very broad transition that is indicative of non-cooperative disruption of the α-helical structure and therefore not amenable to van’t Hoff analysis.

The conformational heterogeneity and apparent dynamic fluctuations of the DAPk-DD give rise to poor dispersion of the resonances in the NMR spectrum (Figure 4A) that preclude direct determination of its structure. CD analysis showed that the DAPk-DD secondary structure remained largely unaffected at different pH values in the presence of 150 mM salt (Figure S5A). However, in the absence of salt and at pH 2 there was a significant loss of DAPk-DD(L) secondary structure manifested by the reduction in ellipticity at 222 nm and the shift of the 207 nm minimum towards 200 nm, indicative of a random coil structure (Figure S5B). Such behavior is consistent with acid denaturation. Varying the ionic strength or adding the detergent SDS had minimal effect on the secondary structure content (Figure S5C).

The addition of 2,2,2-trifluoroethanol (TFE) as a cosolvent has been shown to shift the conformational equilibrium of unfolded or molten globule-like proteins toward a stably-folded conformation suitable for NMR structural analysis [42], [43], [44]. The CD spectrum of both GB1-DAPk-DD(S) and (L) constructs responded to the addition of TFE in a similar manner (Figure 3A), whereas the GB1-FADD-DD construct showed noticeably smaller changes. Changes in the spectra were observed up to a TFE concentration of 40% v/v. The amplitude of the CD signal for the DAPk-DD at 40% v/v TFE was 57% higher (more negative) at 222 nm (−15,951 deg.cm^2^.dmol^−1^) than in the absence of TFE, but lower when compared with the signal for the FADD-DD (∼−25,000 deg cm^2^ dmol^−1^). The addition of 40% v/v TFE to the GB1-FADD-DD construct gave rise to a modest signal increase of 19% at 222 nm, suggesting that the helical content of this protein is closer to the maximum ‘capacity’ of the polypeptide sequence than for the DAPk-DD. The 2D ^1^H-^15^N HSQC spectrum of GB1-DAPk-DD(L) did not show any major change in signal dispersion in the presence of 40% v/v TFE (Figure 4B). There was a slight decrease in resonance line widths and a minor increase in signal dispersion, but without significant perturbation of the distribution of resonances between the ‘dispersed’ and ‘non-dispersed’ subsets. Taken together these observations suggest that TFE has little influence on the overall stability and ‘foldedness’ of the GB1 domain (from which the ‘dispersed’ cross peak subset derives), and likely only stabilizes nascent helical regions of DAPk-DD without the inducement of a stable globular structure.

### Full length-DAPk-DD Interacts with ERK2, without Change in DAPk-DD Secondary Structure

**Figure 5:**
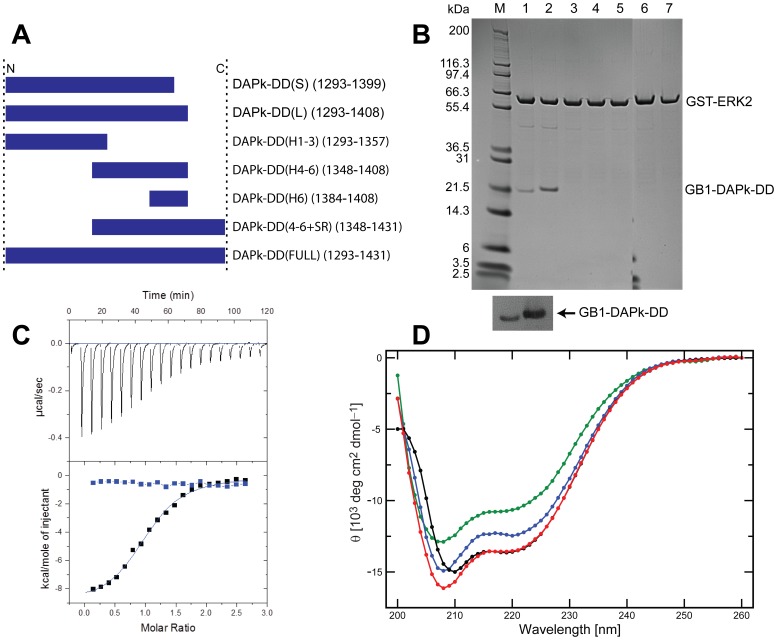
The DAPk-DD interacts with ERK2 and the interaction does not lead to any significant change in the secondary structure of the DAPk-DD. (A) Schematic illustrating the various DAPk-DD peptides used for the ERK2 binding study. (B) LDS-PAGE analysis of the GST pull-down assay showing eluates of GST-ERK2 bound glutathione beads after being incubated with the various GB1-DAPk-DD constructs shown in (A). Lane M, molecular weight size markers (kDa); Lane 1, GB1-DAPk-DD(S); Lane 2, GB1-DAPk-DD(L); Lane 3, GB1-DAPk(H1-3); Lane 4, GB1-DAPk(H4-6); Lane 5, GB1-DAPk(H6); Lane 6, GB1-DAPk(H4-6+SR); Lane 7, GB1-DAPk-DD+SR. Anti-His6 Western blot of the eluates shown in the LDS-PAGE for the short and long GB1-DAPk-DD constructs are shown below the LDS-PAGE. (C) ITC data of GB1-DAPK-DD(L) injected into a solution of ERK2. The upper box shows heat liberated following 19 equal volume injections of GB1-DAPk-DD(L) into 15 µM of ERK2 at 25°C. The integrated heats (lower box) are shown as examples of data quality. The continuous lines represent an optimal fit. For these data, the optimal fit for the DD gives an observed K_d_ of 0.92 µM with an observed *ΔH* of −38.43 kJ/mol. The blue symbols represent the heat of dilution of the control experiment. (D) CD analysis of the ERK-2-DAPk-DD complex. Far-UV CD spectra of ERK2 (blue circles) and GB1-corrected DAPk-DD(L) (green circles) are presented. The signal for the equimolar complex of GB1-DAPk-DD(L) and ERK2 was recorded (black circles), from which the ellipticity of GB1 was subtracted to give the native DAPk-DD-ERK2 CD signal. By combining the individual ERK2 and DAPK-DD CD data (red circles) a very similar CD profile was obtained to that observed for the complex, indicating minor changes in secondary structure to either protein upon formation of a complex. In particular, the signature α-helix wavelength, 222 nm, showed negligible differences. The y-axis represents mean reside ellipticity values and data collection were terminated at 195 nm because of the high tension voltage (dynode) registered at lower wavelengths. The inset represents the data over the wavelength range of 200−230 nm.

It has been reported that the putative DAPk-DD interacts with the extracellular signal-regulated kinases-1 and -2 (ERK 1/2) leading to modulation of the apoptotic activity of DAPk [19]. GST pull-down analysis has shown that a GST-fused DAPk-DD can directly interact with ERK2 via the “DEJL motif” (**RR**DAAD**LLL**) located within a region corresponding to H6 of the putative DAPk-DD fold [19]. Mutation of the DEJL motif hampers the DAPk-DD/ERK2 interaction *in vitro* [19]. To further characterize this protein-protein interaction, we prepared a number of N- and C-terminally truncated GB1-DAPk-DD constructs (Figure 5A) in an effort to identify the minimal ERK2-binding epitope of DAPk-DD. We selected ERK2 over ERK1 because structural and biochemical aspects of this molecule have been analyzed in detail, including its association with the DED-containing phosphoprotein enriched in astrocytes-15 kDa (PEA-15) [45], [46]. The results of the GST pull-down assays showed that, in our hands, only the full-length GB1-DAPk-DD(S) and (L) constructs demonstrated binding to ERK2 in this format (Figure 5B, lanes 1 and 2). Notably neither the GB1-fusion that contains just the DAPk-DD helix-6 (GB1-DAPk-H6) nor the fusion protein containing the C-terminal ‘half’ of DAPk-DD (the H4−H6 construct), both of which include the DEJL motif, were pulled down by GST-ERK2 (Figure 5B, lanes 4 and 5). Consequently, residue(s) within the N-terminal half of the DAPk-DD must also be required to support binding to ERK2. However any such additional ERK2-interacting region(s) within the first three putative helices of the DAPk-DD are not capable of sustaining an interaction with ERK2 on their own, since GST-ERK2 does not pull down GB1-DAPk(H1−H3) (Figure 5B, lane 3). Taken together these results suggest that regions of DAPk-DD outside of the DEJL motif and possibly spread throughout the DD sequence are required for a productive interaction with ERK2.

Unfortunately, the effect of the SR tail of DAPk towards ERK2 binding could not be fully investigated, because the constructs containing the SR tail (Figure 5A) were expressed mainly in the insoluble fraction (data not shown). Nonetheless, crude lysates of these SR tail-containing constructs with appreciably lower amounts of soluble protein compared with the GB1-DAPk-DD variants lacking the SR tail were incubated with GST-ERK2-bound glutathione beads to determine if they would be pulled down with GST-ERK2. The SDS-PAGE analysis (Figure 5B) as well as Western blotting (data not shown) did not reveal any band corresponding to the size of the SR-containing constructs. We conclude that the SR tail is not required for the interaction of the C-terminal region of DAPk with ERK2.

ITC experiments were performed to further characterize the interaction between the DAPk-DD and ERK2 (Figure 5C). Titration of the GB1-DAPk-DD(L) and (S) constructs into ERK2 yielded equilibrium dissociation constants of *K*_d_ = 0.92 µM and 1.77 µM, respectively. In each case the observed stoichiometry (*n*) of the interaction is approximately 1∶1 (*n* = 1.03±0.01 (L) and 0.97±0.01 (S)). The corresponding Δ*H*, *TΔS* and Δ*G* values were −38.4 and −53.1 kJ mol^−1^, −4.0 and −20.3 kJ mol^−1^, and −34.4 and −32.8 kJ mol^−1^, for (L) and (S) constructs respectively. The isolated GB1 domain does not interact with ERK2 (data not shown). These results are consistent with the *in vitro* pull-down experiments showing that GB1-DAPk-DD(S) binds to ERK2 with an apparently lower affinity compared with the GB1-DAPk-DD(L) construct (Figure 5B).

It is conceivable that the interaction could promote ordering of the DAPk-DD polypeptide to a stable α-helix fold. CD spectroscopic analysis was performed to determine whether the interaction of GB1-DAPk-DD with ERK2 induces conformational change. The spectra shown in Figure 5D (and with errors in Figure S6) indicate that the interaction of GB1-DAPk-DD(L) with ERK2 does not lead to any detectable change in the CD response attributable to the GB1-DAPk-DD(L) protein. The ITC and CD results indicate that DAPk-DD is able to bind ERK2 without the induction of a significant degree of additional structural order.

## Discussion

The death domain-region of DAPk is purported to possess an important functional role in the regulation of DAPk catalytic activity [3], [4], [7], [21], [22]. However, no structural characterization of this domain has been reported. In this study, we attempted to examine the structure and oligomeric state of the DAPk-DD. The results of the analysis indicate that, when expressed as a separate protein, this DD does not adopt the stable tertiary structure that has been observed for several other DDs. Rather we found that the protein was poorly behaved and that it was a requirement to append the DAPk-DD with a solubility enhancement tag (here the bacterial protein G B1 domain) in order to recover protein sufficiently tractable for biophysical analysis. Although the GB1-DAPk-DD fusion proteins were soluble, size exclusion chromatography analysis showed that the DAPk-DD fusion constructs exhibited a tendency to reversible self-association, and that this was attributable to the DAPk-DD component. The GB1 domain is known to be highly stable and, as evidenced by the various spectroscopic data obtained for the GB1-DAPk-DD fusion protein, does not interfere with the properties of the appended polypeptide. For example, the dispersed resonances in the 2D ^1^H-^15^N HSQC spectrum of GB1-DAPk-DD correspond precisely with those of the isolated GB1 domain (Figure 4A). Moreover we found no evidence for the thermal ‘melting’ of the GB1 domain in temperature-dependent CD and fluorescence measurements, or any major influence of added TFE co-solvent on the GB1 domain. The thermal mid-point of isolated GB1 had been reported elsewhere to be in the region of ∼360 K [25], well outside the region of interest with respect to the DD component of the fusion proteins (e.g., the FADD-DD component of GB1-FADD-DD unfolds at ∼335 K). The GB1-subtracted CD data indicated that the DAPk-DD component of the GB1-DAPk-DD fusion proteins possess much lower α-helical content than expected for a typical folded DD protein (e.g., 39% for DAPk-DD(L) *vs*. 72% for FADD-DD). The 2D ^1^H-^15^N HSQC spectrum shows very limited chemical shift dispersion for the great majority of resonances not attributable to the GB1 domain, suggesting an absence of globular structure for the DAPk-DD. Consistent with this observation we failed to detect a cooperative unfolding transition, similar to that observed for other GB1-proteins including the GB1-FADD-DD, during temperature-dependent thermal melting experiments monitored by fluorescence. Moreover the high level of Sypro Orange fluorescence observed in this experiment at the starting (low) temperature is consistent with the DAPk-DD protein component lacking ordered structure under ambient conditions. Therefore despite the essentially unambiguous bioinformatic assignment of a death domain fold for the DAPk-DD amino acid sequence, which appears both by visual inspection and quantitative measures of sequence similarity to sit well with other structurally-validated folded death domain proteins (e.g., Figure 1) our data clearly indicate that adoption of a stable globular DD-fold may require one or more factors not present in the polypeptide sequence of this portion of DAPk alone. It is conceivable that, within the holo-DAPk protein, the DAPk-DD is stabilized by interactions with either another part of the (intact) protein chain, an obligate post-translational modification of DAPk, or a constitutive binding partner of DAPk. If this is the case then it would represent a novel instance of a DD that, for stable folding, requires contacts with more or less remote parts of its parent protein and/or an associated polypeptide.

The oligomerization that we observed for the bacterially expressed GB1-DAPk-DD proteins is reminiscent of the reported behavior of a 3*FLAG/*myc*-tagged DAPk-DD construct expressed in HCT116wt cells, or expressed as a His-tagged miniprotein, described by Stevens *et al.* [47]. These authors demonstrated that in the former case the wild-type death domain protein could be chemically cross-linked using ethylene glycol bis(succinimidylsuccinate) (EGS). In the latter case the purified protein eluted by gel filtration in a concentration-dependent manner mostly at ∼600 kDa (assessed by the Bradford assay). A variant form of the DAPk-DD that includes the substitution Asn1347Ser and corresponds to a form of DAPk that lacks the ability to bind ERK2, failed to demonstrate EGS-dependent cross-linking in cells, and as a bacterially expressed protein elutes mostly as a ∼150 kDa species. That the His-tagged protein appears to be tractable as a soluble protein, whereas we found that solubility required the attachment of the GB1 domain, must be attributable to the characteristics of the additional native and non-native vector (pDEST17)-derived N- and C-terminal appendage(s). Unfortunately, from the perspective of this work these authors did not provide any further spectroscopic or structural data for the expressed proteins.

Despite the apparent lack of stable folding, we could demonstrate that the GB1-DAPk-DD(S) and (L) constructs are able to bind ERK2, consistent with reports that have also shown evidence for ERK2/DAPk-DD interactions both *in vivo* and *in vitro* [19],[47]. Our results suggest that additional parts of the DAPk-DD sequence, outside of the DEJL motif, are required for productive interaction. This observation is consistent with the report by Stevens et al. that the Asn1347Ser mutation, corresponding to a site at the C-terminal end of the consensus death domain helix-3, ablates ERK2 binding [47]. We characterized the equilibrium dissociation constants for the complex formed between GST-ERK2 and GB1-DAPk-DD(S) and (L) using microcalorimetry. The low single-digit micromolar *K*_d_ values fall within the range of previously reported protein-ERK interactions. For example the *K*_d_ between PEA-15 and ERK1/2 was found to be 0.2–0.4 µM as assessed by fluorescence anisotropy measurements [48]. For both the GB1-DAPk-DD(S) and (L) constructs, the ITC results indicate a considerable favorable enthalpic contribution (Δ*H*) towards ERK2-binding, suggesting an energy gain from the formation of intra- and/or intermolecular van der Waals contacts. This is accompanied by a significantly unfavorable entropic contribution (*T*Δ*S* is negative) that, in combination with the relatively high exothermic Δ*H* values, is consistent with either the loss of a degree of flexibility or, more generally, a change in the conformation of one or both of the components in the DAPk-DD/ERK2 interaction.

It is tempting to imagine that ERK2-binding might induce ordering of the DAPk-DD polypeptide, perhaps so far as to induce the formation of a canonical six-helix DD fold. We sought evidence for such an induced folding transition by recording CD spectra of the GST-ERK2/GB1-DAPk-DD(L) complex. However, to a good approximation the spectrum of the complex is essentially identical to the sum of the CD spectra of the individual components (Figure 5D), suggesting at most a rather minor conformational change accompanies binding and that the DAPk-DD can bind ERK2 in its apparently non-globular state.

The interaction of ERK1/2 with peptide ligands has been investigated in some detail [49], [50], [51], [52]. ERK1/2 share with other MAP kinases a region referred to as the D-recruitment site (DRS) that interacts with peptide epitopes that contain the canonical sequence (R/K)_2-3_-X_2-6_-Φ_A_-X-Φ_B_, where Φ_A_ and Φ_B_ are hydrophobic residues (typically Leu) [49], [50], [51], [52]. Peptides exhibiting these properties are denoted as ‘D-sites’ or ‘D-motifs’; the ‘D’ refers to an earlier description of the motif as ‘DEJL’ or docking site for ERK, JNK and LXL. Crystal structures of MAP kinases bound to D-motif peptides show that the Φ_A_-X-Φ_B_ portion of the ligand occupies a hydrophobic groove and the positively charged Arg/Lys residues interact with a negatively charged patch called the CD site [53], [54], [55], [56]. However ERK2-directed NMR studies have suggested that ‘non-canonical’ Φ_A_-X-Φ_B_ ligands can bind without engagement of these charged residues [57]. Recently, a structural study has suggested that while these docking nodules of the D-motifs serve to anchor the peptide to MAP kinases, it is the length and amino acid composition of the intervening region between anchor points that are primarily responsible for specificity [54]. ERK1/2 are also reported to possess a second peptide binding site denoted the F-recruitment site (FRS), that is occupied by ‘F-site’ or ‘F-motif’ peptides, first identified as containing the sequence Phe-X-Phe-Pro (FXFP) [58], but now accepted as any similar sequence where the Phe residues are substituted by Tyr or Trp [59]. To date there is no 3D structural data for this latter type of interaction, though the FRS has been mapped to a site distinct from the DRS on the C-terminal lobe of the kinase domain [58].

ERK2 binding to the DED-containing protein PEA-15 has been investigated in some detail [45], [48], [60]. By consensus the PEA-15 binding site mainly includes the ‘RxDLφ-motif' residues on helix H6, the loops connecting helices H1 and H2 and helices H5 and H6, and residues in the flexible C-terminal tail [45], [61]. Curiously the C-terminal tail of PEA-15 contains the sequence I^121^-K-L-A-P-P-P-K-K^129^ which reads like a consensus D-motif peptide ligand except in reverse order. Indeed a variety of measurements suggests that this region of PEA-15 does bind to the ERK2 DRS [61], and that the globular DED part of the protein interacts with a subsidiary site on the kinase domain [45]. Intriguingly recently published heteronuclear NMR relaxation data purporting to describe the ERK2-bound form of PEA-15 have been interpreted in terms of a picture in which the dynamics of the DED domain are strongly perturbed in a non-uniform manner, leading the authors to ascribe an induced-fit mechanism and suggesting a degree of plasticity in the PEA-15 DED domain [62]. The picture that emerges is one in which the ERK1/2 kinase domain interacts with regulatory ligands that lack a high degree of structural order.

How does this picture of ERK-ligand interactions apply to our observations with DAPk-DD? Our data clearly suggest that DAPk-DD does not function as a canonical death domain fold in order to bind to ERK2. This might suggest that the DAPk-DD could bind ERK2 via either the DRS or FRS, or both. Inspection of the amino acid sequence of the DAPk-DD constructs that we employed shows that: the R^1311^-R-K-L-S-R-L-L-D^1319^ sequence near the N-terminus comes close to matching the D-motif consensus but lacks a hydrophobe at the Φ_B_ residue (Φ_B_ = Asp); the R^1312^-K-L-S-R-L-L-D-P^1320^ sequence is a close but non-exact match (Φ_B_ = Pro) to the D-site consensus; separately, Φ_A_-X-Φ_B_ D-‘subsite’ motifs occur at L^1297^-G-M^1299^, F^1354^-L-P^1356^, L^1392^-L-L^1394^ and I^1402^-N-L^1404^; and the sequence does not contain any amino acid stretch that is a close match to the F-site consensus. Thus the observed binding of DAPk-DD to ERK2 is likely attributable, at least in part, to an occupation of the DRS by one of the (non-exact) D-motifs that would be available within a disordered form of the DAPk-DD polypeptide. In fact, in the absence of an induced-fit mechanism, a globular DD-fold would, by homology, sequester the critical Φ_A_-X-Φ_B_ hydrophobic residues in the core of the domain. However, neither the N- or C-terminal truncation mutants of DAPk-DD are competent to bind ERK2, therefore suggesting that binding must involve additional contacts outside of the putative DRS/D-motif interaction. This is reminiscent of the conclusions drawn for PEA-15 which is suggested to bind ERK2 in a ‘bidentate’ fashion [48], [61].

In summary we have provided evidence that the death domain of DAPk is not stably folded in a classical, globular α-helical form, at least when expressed by itself out of the context of its ‘host’ *holo-*polypeptide. However, even in its partially helical, but mostly disordered, state the DAPk-DD is able to bind ERK2 with low micromolar affinity. Even though the binding does not involve induced folding of the DAPk-DD, it appears to require elements of the both the N- and C-terminal stretches of the protein. Collectively, these observations provide a platform for further investigation of the regulation of ERK1/2 and DAPk kinase activity in cell survival and death pathways at the molecular, cellular and biochemical level. Moreover emerging progress into the structural characterization of ERK2 by frontier methods in NMR spectroscopy [57], [63] promise an opportunity to explore the structural basis for its interaction with DAPk at the atomic scale. Moreover it will be intriguing to explore whether under the conditions within the cell, that include the consequences of molecular crowding on protein stability, the folding behavior of DAPk-DD is altered, and in addition the folding status of the DAPk-DD polypeptide in interactions either *in vitro* or *in vivo* with other purported binding partners such as the netrin-1 receptor UNC5H2.

## Supporting Information

Figure S1 **Protein purification by size-exclusion chromatography and analysis of the collected fractions by SDS-PAGE. (**A) GB1-FADD-DD, GB1-DAPk-DD(S) and GB1-DAPk-DD(L) were purified by SEC on a Superdex 75 column (GE Healthcare) under similar conditions. The overlaid chromatograms clearly show a difference in profiles between GB1-FADD-DD and the two GB1-DAPk-DD constructs, as well as a difference in the elution volumes. The elution volume of the first peak is similar to the void volume and represents aggregated DAPk-DD species >70 kDa that do not dissociate under non-denaturing conditions. The second major peak represents oligomeric GB1-DAPk-DD species, which readily undergo reversible dissociation upon dilution to lower concentrations (see the Results section for further details and **(C)**). As a control, GB1-FADD-DD was purified using the same method and elutes at a later elution volume when compared with the elution volumes of the peaks arising from the GB1-DAPk-DD constructs. (**B**) SDS-PAGE analysis of GB1-DAPk-DD(S) fractions collected in (**A**). M: marker (MWs in kDa), A0: Ni/NTA eluate loaded onto the column, A6−A10: SEC fractions taken from (**A**). The gel bands were stained with Coomassie blue dye. (**C**) Affinity and SEC purified GB1-FADD-DD in 20 mM NaPi, 150 mM NaCl, 3 mM DTT, pH 6.2 and GB1-DAPk-DD(S) in 20 mM NaPi, 150 mM NaCl, 3 mM DTT, pH 7.4 were applied on a pre-packed Superose-12 analytical size-exclusion column (Amersham Biosciences) at the same concentration, i.e., 1 mg/ml. Coloured as in A. At a lower concentration, the purified GB1-DAPk-DD(S) protein elutes at an elution volume near identical to the GB1-FADD-DD protein, which does not form oligomers. This result shows that at the loading concentration of 1 mg/ml, the GB1-DAPk-DD(S) construct behaves as a monomer.
(DOCX)

Figure S2 **Elution profiles of proteins studied by analytical size exclusion chromatography.** Overlay of the different concentration runs on a Superose-12 column of ovalbumin (A), GB1-FADD-DD (C), GB1-DAPk-DD(S) (E) and GB1-DAPk-DD(L) (G) and zoomed in regions are presented in B, D, F and H, respectively. AU refers to the instrument absorbance units at 280 nm. Different concentrations in mg/ml from the highest to the lowest are as follow: blue (20), pink (10), red (5), cyan (1), purple (0.5), brown (0.25), green (0.1) and orange (0.05).
(DOCX)

Figure S3 **Analytical gel filtration calibration curve. (A)** Protein markers (Biorad) consisting of thyroglobulin (670 kDa), bovine gamma-globulin (158 kDa), chicken ovalbumin (44 kDa), equine myoglobin (17 kDa) and vitamin B_12_ (1.35 kDa) were run through the Superose-12 column. The order of the markers description follows the order of elution from left to right. The elution volumes of the protein markers, shown in ml next to the corresponding peaks, were calculated with the Unicorn 3.0 software package (Amersham Biosciences). Absorbance measured at 280 nm. Elution volumes related to the MWs (logarithmic scale) of the markers and the formula that correlates them are shown in (**B)**. Diamond-shaped points in **(B)** represent the elution volumes derived in **(A)**.
(DOCX)

Figure S4 **CD spectra of DAPk-DD at 5, 25 and 95°C.** CD spectra of GB1-corrected DAPk-DD(L) at 5°C (blue), 25°C (green) and 95°C (red). The CD spectra show no significant profile differences or signs of protein aggregation. A noticeable reduction in MRE values at the highest temperature is caused by the thermal denaturation of the secondary structure of the DAPk-DD. Error bars are estimates of uncertainties derived from the three scans recorded per sample.
(DOCX)

Figure S5 **GB1-DAPk-DD shows little secondary structure change under different buffer conditions.** Far-UV CD data of the GB1-subtracted native DAPk-DD(L) in different buffers. The effect of extreme pH (red symbols = pH 2; green symbols = pH 7.4; blue symbols = pH 12) on the secondary structure of the DAPk-DD(L) is shown either in the presence of 150 mM NaCl (**A**) or in the absence of NaCl (**B**). Only at pH 2 in the absence of salt (B, red symbols) did the DD show substantial changes in structure. (**C**) At pH 7.4, the influence of the detergent SDS was examined. Only minor differences were seen in the CD spectra recorded on GB1-DAPk-DD(L) samples measured in the absence of SDS (green), 2 mM (red; below CMC) and 50 mM (blue; above CMC) SDS. The result indicates that SDS induced only slight secondary structure changes to the DD both below and above the CMC.
(DOCX)

Figure S6 **The interaction of the DAPk-DD with ERK2 does not lead to any significant change in the secondary structure of the DAPk-DD or ERK2 proteins.** CD analysis of the ERK-2-DAPk-DD complex. Far-UV CD spectra of ERK2 (blue circles) and GB1-corrected DAPk-DD(L) (green circles) are presented. The signal for the equimolar complex of GB1-DAPk-DD(L) and ERK2 was recorded (black circles), from which the ellipticity of GB1 was subtracted to give the native DAPk-DD-ERK2 CD signal. By combining the individual ERK2 and DAPK-DD CD data (red circles) a near identical CD profile was obtained to that observed for the complex, indicating negligible changes in secondary structure to either protein upon formation of a complex. The error bars are estimates of uncertainties derived from the three scans recorded per sample. The inset represents the data over the wavelength range of 200−230 nm.
(DOCX)

## References

[1] Deiss LP, Feinstein E, Berissi H, Cohen O, Kimchi A (1995) Identification of a novel serine/threonine kinase and a novel 15-kD protein as potential mediators of the gamma interferon-induced cell death. Genes Dev 9: 15–30. 7828849 10.1101/gad.9.1.15

[2] Gozuacik D, Bialik S, Raveh T, Mitou G, Shohat G, et al (2008) DAP-kinase is a mediator of endoplasmic reticulum stress-induced caspase activation and autophagic cell death. Cell Death Differ 15: 1875–1886. 18806755 10.1038/cdd.2008.121

[3] Cohen O, Feinstein E, Kimchi A (1997) DAP-kinase is a Ca2+/calmodulin-dependent, cytoskeletal-associated protein kinase, with cell death-inducing functions that depend on its catalytic activity. EMBO J 16: 998–1008. 9118961 10.1093/emboj/16.5.998 PMC1169700

[4] Cohen O, Inbal B, Kissil JL, Raveh T, Berissi H, et al (1999) DAP-kinase participates in TNF-alpha- and Fas-induced apoptosis and its function requires the death domain. J Cell Biol 146: 141–148. 10402466 10.1083/jcb.146.1.141 PMC2199731

[5] Inbal B, Cohen O, Polak-Charcon S, Kopolovic J, Vadai E, et al (1997) DAP kinase links the control of apoptosis to metastasis. Nature 390: 180–184. 9367156 10.1038/36599

[6] Jang CW, Chen CH, Chen CC, Chen JY, Su YH, et al (2002) TGF-beta induces apoptosis through Smad-mediated expression of DAP-kinase. Nat Cell Biol 4: 51–58. 11740493 10.1038/ncb731

[7] Llambi F, Lourenco FC, Gozuacik D, Guix C, Pays L, et al (2005) The dependence receptor UNC5H2 mediates apoptosis through DAP-kinase. EMBO J 24: 1192–1201. 15729359 10.1038/sj.emboj.7600584 PMC556396

[8] Pelled D, Raveh T, Riebeling C, Fridkin M, Berissi H, et al (2002) Death-associated protein (DAP) kinase plays a central role in ceramide-induced apoptosis in cultured hippocampal neurons. J Biol Chem 277: 1957–1961. 11709549 10.1074/jbc.M104677200

[9] Raveh T, Kimchi A (2001) DAP kinase-a proapoptotic gene that functions as a tumor suppressor. Exp Cell Res 264: 185–192. 11237533 10.1006/excr.2000.5134

[10] Yamamoto M, Hioki T, Ishii T, Nakajima-Iijima S, Uchino S (2002) DAP kinase activity is critical for C(2)-ceramide-induced apoptosis in PC12 cells. Eur J Biochem 269: 139–147. 11784307 10.1046/j.0014-2956.2002.00029.x

[11] Raveh T, Berissi H, Eisenstein M, Spivak T, Kimchi A (2000) A functional genetic screen identifies regions at the C-terminal tail and death-domain of death-associated protein kinase that are critical for its proapoptotic activity. Proc Natl Acad Sci USA 97: 1572–1577. 10677501 10.1073/pnas.020519497 PMC26476

[12] Carlessi R, Levin-Salomon V, Ciprut S, Bialik S, Berissi H, et al (2011) GTP binding to the ROC domain of DAP-kinase regulates its function through intramolecular signalling. EMBO Rep 12: 917–923. 21738225 10.1038/embor.2011.126 PMC3166453

[13] Ferrao R, Wu H (2012) Helical assembly in the death domain (DD) superfamily. Curr Opin Struct Biol 22: 241–247. 22429337 10.1016/j.sbi.2012.02.006 PMC3320699

[14] Park HH, Logette E, Raunser S, Cuenin S, Walz T, et al (2007) Death domain assembly mechanism revealed by crystal structure of the oligomeric PIDDosome core complex. Cell 128: 533–546. 17289572 10.1016/j.cell.2007.01.019 PMC2908332

[15] Esposito D, Sankar A, Morgner N, Robinson CV, Rittinger K, et al (2010) Solution NMR investigation of the CD95/FADD homotypic death domain complex suggests lack of engagement of the CD95 C terminus. Structure 18: 1378–1390. 20947025 10.1016/j.str.2010.08.006

[16] Scott FL, Stec B, Pop C, Dobaczewska MK, Lee JJ, et al (2009) The Fas-FADD death domain complex structure unravels signalling by receptor clustering. Nature 457: 1019–1022. 19118384 10.1038/nature07606 PMC2661029

[17] Wang L, Yang JK, Kabaleeswaran V, Rice AJ, Cruz AC, et al (2010) The Fas-FADD death domain complex structure reveals the basis of DISC assembly and disease mutations. Nat Struct Mol Biol 17: 1324–1329. 20935634 10.1038/nsmb.1920 PMC2988912

[18] Lin SC, Lo YC, Wu H (2010) Helical assembly in the MyD88-IRAK4-IRAK2 complex in TLR/IL-1R signalling. Nature 465: 885–890. 20485341 10.1038/nature09121 PMC2888693

[19] Chen CH, Wang WJ, Kuo JC, Tsai HC, Lin JR, et al (2005) Bidirectional signals transduced by DAPK-ERK interaction promote the apoptotic effect of DAPK. EMBO J 24: 294–304. 15616583 10.1038/sj.emboj.7600510 PMC545805

[20] Wu PR, Tsai PI, Chen GC, Chou HJ, Huang YP, et al (2011) DAPK activates MARK1/2 to regulate microtubule assembly, neuronal differentiation, and tau toxicity. Cell Death Differ 18: 1507–1520. 21311567 10.1038/cdd.2011.2 PMC3178434

[21] Stevens C, Lin Y, Harrison B, Burch L, Ridgway RA, et al (2009) Peptide combinatorial libraries identify TSC2 as a death-associated protein kinase (DAPK) death domain-binding protein and reveal a stimulatory role for DAPK in mTORC1 signaling. J Biol Chem 284: 334–344. 18974095 10.1074/jbc.M805165200

[22] Mor I, Carlessi R, Ast T, Feinstein E, Kimchi A (2012) Death-associated protein kinase increases glycolytic rate through binding and activation of pyruvate kinase. Oncogene 31: 683–693. 21725354 10.1038/onc.2011.264

[23] Huth JR, Bewley CA, Jackson BM, Hinnebusch AG, Clore GM, et al (1997) Design of an expression system for detecting folded protein domains and mapping macromolecular interactions by NMR. Protein Sci 6: 2359–2364. 9385638 10.1002/pro.5560061109 PMC2143577

[24] Berglund H, Olerenshaw D, Sankar A, Federwisch M, McDonald NQ, et al (2000) The three-dimensional solution structure and dynamic properties of the human FADD death domain. J Mol Biol 302: 171–188. 10964568 10.1006/jmbi.2000.4011

[25] Alexander P, Fahnestock S, Lee T, Orban J, Bryan P (1992) Thermodynamic analysis of the folding of the streptococcal protein G IgG-binding domains B1 and B2: why small proteins tend to have high denaturation temperatures. Biochemistry 31: 3597–3603. 1567818 10.1021/bi00129a007

[26] Gill SC, von Hippel PH (1989) Calculation of protein extinction coefficients from amino acid sequence data. Anal Biochem 182: 319–326. 2610349 10.1016/0003-2697(89)90602-7

[27] Delaglio F, Grzesiek S, Vuister GW, Zhu G, Pfeifer J, et al (1995) NMRPipe: a multidimensional spectral processing system based on UNIX pipes. J Biomol NMR 6: 277–293. 8520220 10.1007/BF00197809

[28] Vranken WF, Boucher W, Stevens TJ, Fogh RH, Pajon A, et al (2005) The CCPN data model for NMR spectroscopy: development of a software pipeline. Proteins 59: 687–696. 15815974 10.1002/prot.20449

[29] Holm L, Park J (2000) DaliLite workbench for protein structure comparison. Bioinformatics 16: 566–567. 10980157 10.1093/bioinformatics/16.6.566

[30] Larkin MA, Blackshields G, Brown NP, Chenna R, McGettigan PA, et al (2007) Clustal W and Clustal X version 2.0. Bioinformatics 23: 2947–2948. 17846036 10.1093/bioinformatics/btm404

[31] Zhou P, Wagner G (2010) Overcoming the solubility limit with solubility-enhancement tags: successful applications in biomolecular NMR studies. J Biomol NMR 46: 23–31. 19731047 10.1007/s10858-009-9371-6 PMC2879018

[32] Hiller S, Kohl A, Fiorito F, Herrmann T, Wider G, et al (2003) NMR structure of the apoptosis- and inflammation-related NALP1 pyrin domain. Structure 11: 1199–1205. 14527388 10.1016/j.str.2003.08.009

[33] Alfarano C, Andrade CE, Anthony K, Bahroos N, Bajec M, et al (2005) The Biomolecular Interaction Network Database and related tools 2005 update. Nucleic Acids Res 33: D418–D424. 15608229 10.1093/nar/gki051 PMC540005

[34] De Wilde G, Murray-Rust J, Boone E, Olerenshaw D, McDonald NQ, et al (2001) Structure-activity relationship of the p55 TNF receptor death domain and its lymphoproliferation mutants. Eur J Biochem 268: 1382–1391. 11231290 10.1046/j.1432-1327.2001.02004.x

[35] Moncrieffe MC, Stott KM, Gay NJ (2005) Solution structure of the isolated Pelle death domain. FEBS Lett 579: 3920–3926. 16004997 10.1016/j.febslet.2005.06.009

[36] Compton LA, Johnson WC Jr (1986) Analysis of protein circular dichroism spectra for secondary structure using a simple matrix multiplication. Anal Biochem 155: 155–167. 3717552 10.1016/0003-2697(86)90241-1

[37] Johnson WC (1999) Analyzing protein circular dichroism spectra for accurate secondary structures. Proteins 35: 307–312. 10328265

[38] Manavalan P, Johnson WC Jr (1987) Variable selection method improves the prediction of protein secondary structure from circular dichroism spectra. Anal Biochem 167: 76–85. 3434802 10.1016/0003-2697(87)90135-7

[39] Sreerama N, Woody RW (2000) Estimation of protein secondary structure from circular dichroism spectra: comparison of CONTIN, SELCON, and CDSSTR methods with an expanded reference set. Anal Biochem 287: 252–260. 11112271 10.1006/abio.2000.4880

[40] Ericsson UB, Hallberg BM, Detitta GT, Dekker N, Nordlund P (2006) Thermofluor-based high-throughput stability optimization of proteins for structural studies. Anal Biochem 357: 289–298. 16962548 10.1016/j.ab.2006.07.027

[41] Pantoliano MW, Petrella EC, Kwasnoski JD, Lobanov VS, Myslik J, et al (2001) High-density miniaturized thermal shift assays as a general strategy for drug discovery. J Biomol Screen 6: 429–440. 11788061 10.1177/108705710100600609

[42] Buck M (1998) Trifluoroethanol and colleagues: cosolvents come of age. Recent studies with peptides and proteins. Q Rev Biophys 31: 297–355. 10384688 10.1017/s003358359800345x

[43] Chen L, Balabanidou V, Remeta DP, Minetti CA, Portaliou AG, et al (2011) Structural instability tuning as a regulatory mechanism in protein-protein interactions. Mol Cell 44: 734–744. 22152477 10.1016/j.molcel.2011.09.022 PMC3240846

[44] Kim AS, Kakalis LT, Abdul-Manan N, Liu GA, Rosen MK (2000) Autoinhibition and activation mechanisms of the Wiskott-Aldrich syndrome protein. Nature 404: 151–158. 10724160 10.1038/35004513

[45] Hill JM, Vaidyanathan H, Ramos JW, Ginsberg MH, Werner MH (2002) Recognition of ERK MAP kinase by PEA-15 reveals a common docking site within the death domain and death effector domain. EMBO J 21: 6494–6504. 12456656 10.1093/emboj/cdf641 PMC136945

[46] Formstecher E, Ramos JW, Fauquet M, Calderwood DA, Hsieh JC, et al (2001) PEA-15 mediates cytoplasmic sequestration of ERK MAP kinase. Dev Cell 1: 239–250. 11702783 10.1016/s1534-5807(01)00035-1

[47] Stevens C, Lin Y, Sanchez M, Amin E, Copson E, et al (2007) A germ line mutation in the death domain of DAPK-1 inactivates ERK-induced apoptosis. J Biol Chem 282: 13791–13803. 17244621 10.1074/jbc.M605649200

[48] Callaway K, Rainey MA, Dalby KN (2005) Quantifying ERK2-protein interactions by fluorescence anisotropy: PEA-15 inhibits ERK2 by blocking the binding of DEJL domains. Biochim Biophys Acta 1754: 316–323. 16324895 10.1016/j.bbapap.2005.11.002

[49] Bardwell AJ, Frankson E, Bardwell L (2009) Selectivity of docking sites in MAPK kinases. J Biol Chem 284: 13165–13173. 19196711 10.1074/jbc.M900080200 PMC2676048

[50] Sharrocks AD, Yang SH, Galanis A (2000) Docking domains and substrate-specificity determination for MAP kinases. Trends Biochem Sci 25: 448–453. 10973059 10.1016/s0968-0004(00)01627-3

[51] Tanoue T, Maeda R, Adachi M, Nishida E (2001) Identification of a docking groove on ERK and p38 MAP kinases that regulates the specificity of docking interactions. EMBO J 20: 466–479. 11157753 10.1093/emboj/20.3.466 PMC133461

[52] Zhou B, Wu L, Shen K, Zhang J, Lawrence DS, et al (2001) Multiple regions of MAP kinase phosphatase 3 are involved in its recognition and activation by ERK2. J Biol Chem 276: 6506–6515. 11104775 10.1074/jbc.M009753200

[53] Chang CI, Xu BE, Akella R, Cobb MH, Goldsmith EJ (2002) Crystal structures of MAP kinase p38 complexed to the docking sites on its nuclear substrate MEF2A and activator MKK3b. Mol Cell 9: 1241–1249. 12086621 10.1016/s1097-2765(02)00525-7

[54] Garai A, Zeke A, Gogl G, Toro I, Fordos F, et al (2012) Specificity of linear motifs that bind to a common mitogen-activated protein kinase docking groove. Sci Signal 5: ra74. 23047924 10.1126/scisignal.2003004 PMC3500698

[55] Heo YS, Kim SK, Seo CI, Kim YK, Sung BJ, et al (2004) Structural basis for the selective inhibition of JNK1 by the scaffolding protein JIP1 and SP600125. EMBO J 23: 2185–2195. 15141161 10.1038/sj.emboj.7600212 PMC419904

[56] Liu S, Sun JP, Zhou B, Zhang ZY (2006) Structural basis of docking interactions between ERK2 and MAP kinase phosphatase 3. Proc Natl Acad Sci USA 103: 5326–5331. 16567630 10.1073/pnas.0510506103 PMC1459354

[57] Piserchio A, Warthaka M, Devkota AK, Kaoud TS, Lee S, et al (2011) Solution NMR insights into docking interactions involving inactive ERK2. Biochemistry 50: 3660–3672. 21449613 10.1021/bi2000559 PMC3103835

[58] Lee T, Hoofnagle AN, Kabuyama Y, Stroud J, Min X, et al (2004) Docking motif interactions in MAP kinases revealed by hydrogen exchange mass spectrometry. Mol Cell 14: 43–55. 15068802 10.1016/s1097-2765(04)00161-3

[59] Sheridan DL, Kong Y, Parker SA, Dalby KN, Turk BE (2008) Substrate discrimination among mitogen-activated protein kinases through distinct docking sequence motifs. J Biol Chem 283: 19511–19520. 18482985 10.1074/jbc.M801074200 PMC2443660

[60] Whitehurst AW, Robinson FL, Moore MS, Cobb MH (2004) The death effector domain protein PEA-15 prevents nuclear entry of ERK2 by inhibiting required interactions. J Biol Chem 279: 12840–12847. 14707138 10.1074/jbc.M310031200

[61] Callaway K, Abramczyk O, Martin L, Dalby KN (2007) The anti-apoptotic protein PEA-15 is a tight binding inhibitor of ERK1 and ERK2, which blocks docking interactions at the D-recruitment site. Biochemistry 46: 9187–9198. 17658892 10.1021/bi700206u

[62] Twomey EC, Cordasco DF, Wei Y (2012) Profound conformational changes of PED/PEA-15 in ERK2 complex revealed by NMR backbone dynamics. Biochim Biophys Acta 1824: 1382–1393. 22820249 10.1016/j.bbapap.2012.07.001

[63] Piserchio A, Dalby KN, Ghose R (2012) Assignment of backbone resonances in a eukaryotic protein kinase - ERK2 as a representative example. Methods Mol Biol 831: 359–368. 22167683 10.1007/978-1-61779-480-3_19 PMC3305997

