# Supplementary material for: Structural and Functional Characterization of the Recombinant Death Domain from Death-Associated Protein Kinase
Source: PLoS One. 2013 Jul 29;8(7):e70095. doi: 10.1371/journal.pone.0070095 (PMC3726526; doi:10.1371/journal.pone.0070095)
Supplement: Figure S1 — Protein purification by size-exclusion chromatography and analysis of the collected fractions by SDS-PAGE. (A) GB1-FADD-DD, GB1-DAPk-DD(S) and GB1-DAPk-DD(L) were purified by SEC on a Superdex 75 column (GE Healthcare) under similar conditions. The overlaid chromatograms clearly show a difference in profiles between GB1-FADD-DD and the two GB1-DAPk-DD constructs, as well as a difference in the elution volumes. The elution volume of the first peak is similar to the void volume and represents aggregated DAPk-DD species >70 kDa that do not dissociate under non-denaturing conditions. The second major peak represents oligomeric GB1-DAPk-DD species, which readily undergo reversible dissociation upon dilution to lower concentrations (see the Results section for further details and (C)). As a control, GB1-FADD-DD was purified using the same method and elutes at a later elution volume when compared with the elution volumes of the peaks arising from the GB1-DAPk-DD constructs. (B) SDS-PAGE analysis of GB1-DAPk-DD(S) fractions collected in (A). M: marker (MWs in kDa), A0: Ni/NTA eluate loaded onto the column, A6−A10: SEC fractions taken from (A). The gel bands were stained with Coomassie blue dye. (C) Affinity and SEC purified GB1-FADD-DD in 20 mM NaPi, 150 mM NaCl, 3 mM DTT, pH 6.2 and GB1-DAPk-DD(S) in 20 mM NaPi, 150 mM NaCl, 3 mM DTT, pH 7.4 were applied on a pre-packed Superose-12 analytical size-exclusion column (Amersham Biosciences) at the same concentration, i.e., 1 mg/ml. Coloured as in A. At a lower concentration, the purified GB1-DAPk-DD(S) protein elutes at an elution volume near identical to the GB1-FADD-DD protein, which does not form oligomers. This result shows that at the loading concentration of 1 mg/ml, the GB1-DAPk-DD(S) construct behaves as a monomer. (DOCX) [file pone.0070095.s001.docx]

**(A)**


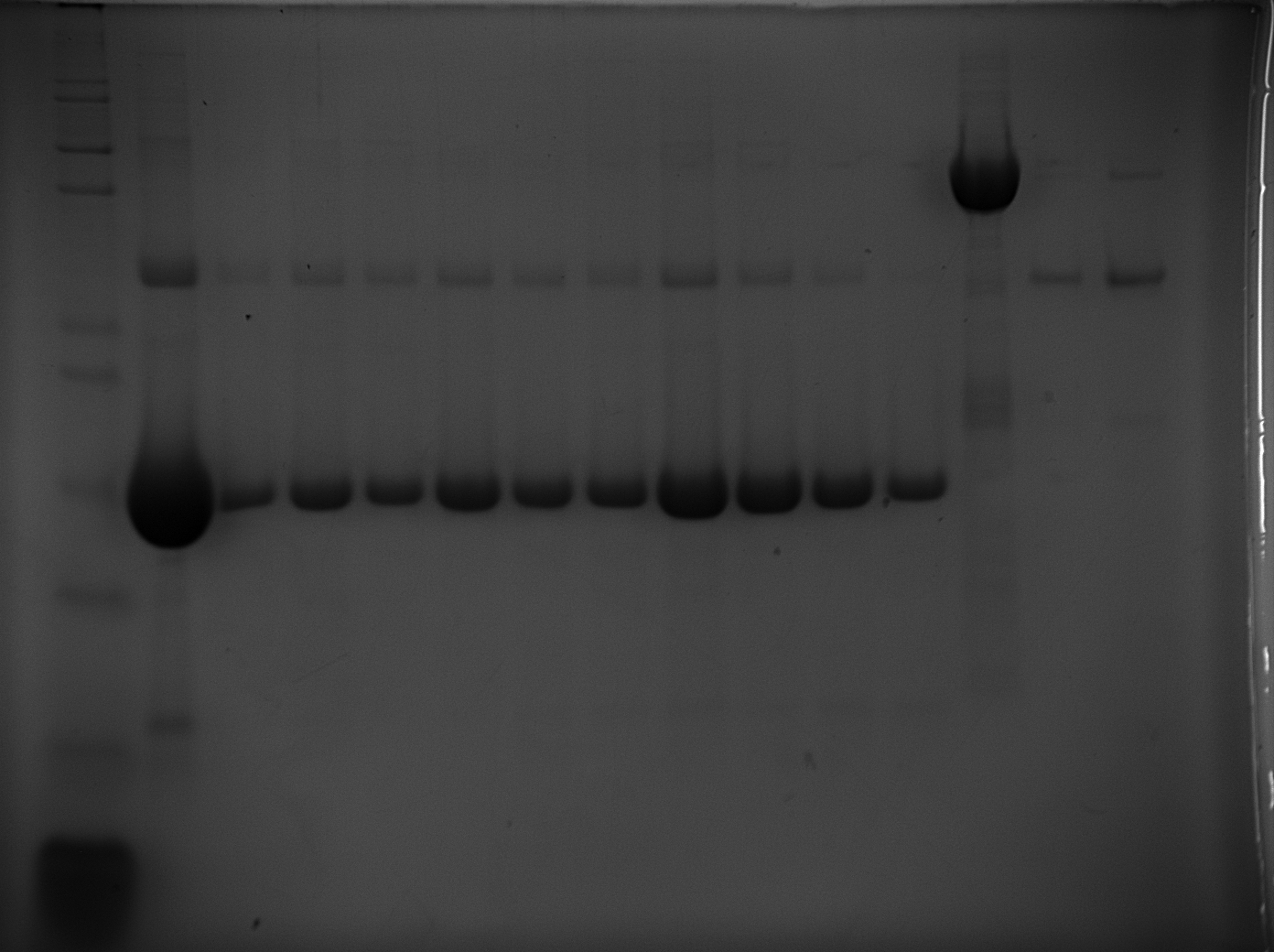


M A0 A6 A7 A8 A9 A10

**(B).**

2.5

3.5

6

14.4

21.5

31

36.5

55.4

66.3


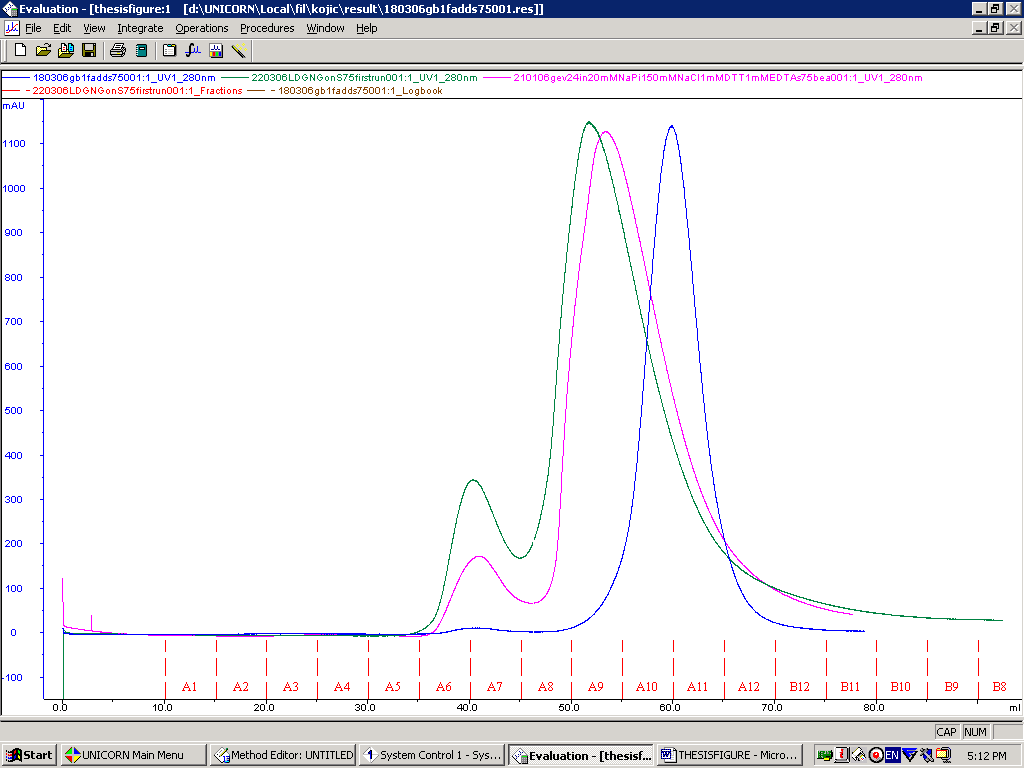


10

30

40

50

60

70

80

Elution volume (ml)

A1

A2

A3

A4

A5

A6

A7

A8

A9

A10

A11

A12

B12

B11

B10

B9

B8

20

0

200

400

600

800

1000

GB1-FADD-DD

GB1-DAPk-DD(S)

GB1-DAPk-DD(L)


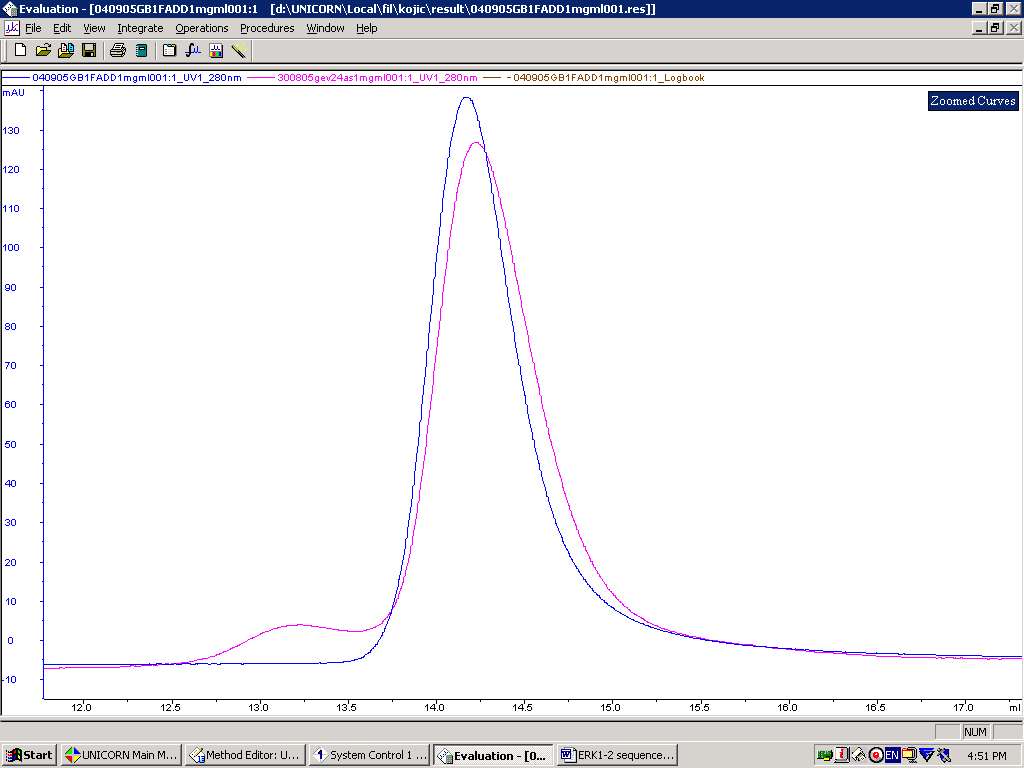

13.0

13.5

14.0

14.5

15.0

15.5

16.0

16.5

17.0

Absorbance (280 nm)

Elution volume (ml)

**A.**

**(C)**

Absorbance

(280 nm)

**Figure S1. Protein purification by size-exclusion chromatography and analysis of the collected fractions by SDS-PAGE. (**A) GB1-FADD-DD, GB1-DAPk-DD(S) and GB1-DAPk-DD(L) were purified by SEC on a Superdex 75 column (GE Healthcare) under similar conditions. The overlaid chromatograms clearly show a difference in profiles between GB1-FADD-DD and the two GB1-DAPk-DD constructs, as well as a difference in the elution volumes. The elution volume of the first peak is similar to the void volume and represents aggregated DAPk-DD species >70 kDa that do not dissociate under non-denaturing conditions. The second major peak represents oligomeric GB1-DAPk-DD species, which readily undergo reversible dissociation upon dilution to lower concentrations (see the Results section for further details and **(C)**). As a control, GB1-FADD-DD was purified using the same method and elutes at a later elution volume when compared with the elution volumes of the peaks arising from the GB1-DAPk-DD constructs. (**B**) SDS-PAGE analysis of GB1-DAPk-DD(S) fractions collected in (**A**). M: marker (MWs in kDa), A0: Ni/NTA eluate loaded onto the column, A6−A10: SEC fractions taken from (**A**). The gel bands were stained with Coomassie blue dye. (**C**) Affinity and SEC purified GB1-FADD-DD in 20 mM NaPi, 150 mM NaCl, 3 mM DTT, pH 6.2 and GB1-DAPk-DD(S) in 20 mM NaPi, 150 mM NaCl, 3 mM DTT, pH 7.4 were applied on a pre-packed Superose-12 analytical size-exclusion column (Amersham Biosciences) at the same concentration, i.e., 1 mg/ml. Coloured as in A. At a lower concentration, the purified GB1-DAPk-DD(S) protein elutes at an elution volume near identical to the GB1-FADD-DD protein, which does not form oligomers. This result shows that at the loading concentration of 1 mg/ml, the GB1-DAPk-DD(S) construct behaves as a monomer.
