# Supplementary material for: Structural and Functional Characterization of the Recombinant Death Domain from Death-Associated Protein Kinase
Source: PLoS One. 2013 Jul 29;8(7):e70095. doi: 10.1371/journal.pone.0070095 (PMC3726526; doi:10.1371/journal.pone.0070095)
Supplement: Figure S2 — Elution profiles of proteins studied by analytical size exclusion chromatography. Overlay of the different concentration runs on a Superose-12 column of ovalbumin (A), GB1-FADD-DD (C), GB1-DAPk-DD(S) (E) and GB1-DAPk-DD(L) (G) and zoomed in regions are presented in B, D, F and H, respectively. AU refers to the instrument absorbance units at 280 nm. Different concentrations in mg/ml from the highest to the lowest are as follow: blue (20), pink (10), red (5), cyan (1), purple (0.5), brown (0.25), green (0.1) and orange (0.05). (DOCX) [file pone.0070095.s002.docx]

**H**

**G**

(ml)

(ml)

**A**

**B**

AU 280 nm

AU 280 nm

**C**

**D**

**E**

**F**

(ml)

(ml)

(ml)

(ml)

(ml)

(ml)

AU 280 nm

AU 280 nm

AU 280 nm

AU 280 nm

AU 280 nm

AU 280 nm

**Figure S2.** **Elution profiles of proteins studied by analytical size exclusion chromatography.** Overlay of the different concentration runs on a Superose-12 column of ovalbumin (A), GB1-FADD-DD (C), GB1-DAPk-DD(S) (E) and GB1-DAPk-DD(L) (G) and zoomed in regions are presented in B, D, F and H, respectively. AU refers to the instrument absorbance units at 280 nm. Different concentrations in mg/ml from the highest to the lowest are as follow: blue (20), pink (10), red (5), cyan (1), purple (0.5), brown (0.25), green (0.1) and orange (0.05).
