# Supplementary material for: Structural and Functional Characterization of the Recombinant Death Domain from Death-Associated Protein Kinase
Source: PLoS One. 2013 Jul 29;8(7):e70095. doi: 10.1371/journal.pone.0070095 (PMC3726526; doi:10.1371/journal.pone.0070095)
Supplement: Figure S3 — Analytical gel filtration calibration curve. (A) Protein markers (Biorad) consisting of thyroglobulin (670 kDa), bovine gamma-globulin (158 kDa), chicken ovalbumin (44 kDa), equine myoglobin (17 kDa) and vitamin B12 (1.35 kDa) were run through the Superose-12 column. The order of the markers description follows the order of elution from left to right. The elution volumes of the protein markers, shown in ml next to the corresponding peaks, were calculated with the Unicorn 3.0 software package (Amersham Biosciences). Absorbance measured at 280 nm. Elution volumes related to the MWs (logarithmic scale) of the markers and the formula that correlates them are shown in (B). Diamond-shaped points in (B) represent the elution volumes derived in (A). (DOCX) [file pone.0070095.s003.docx]

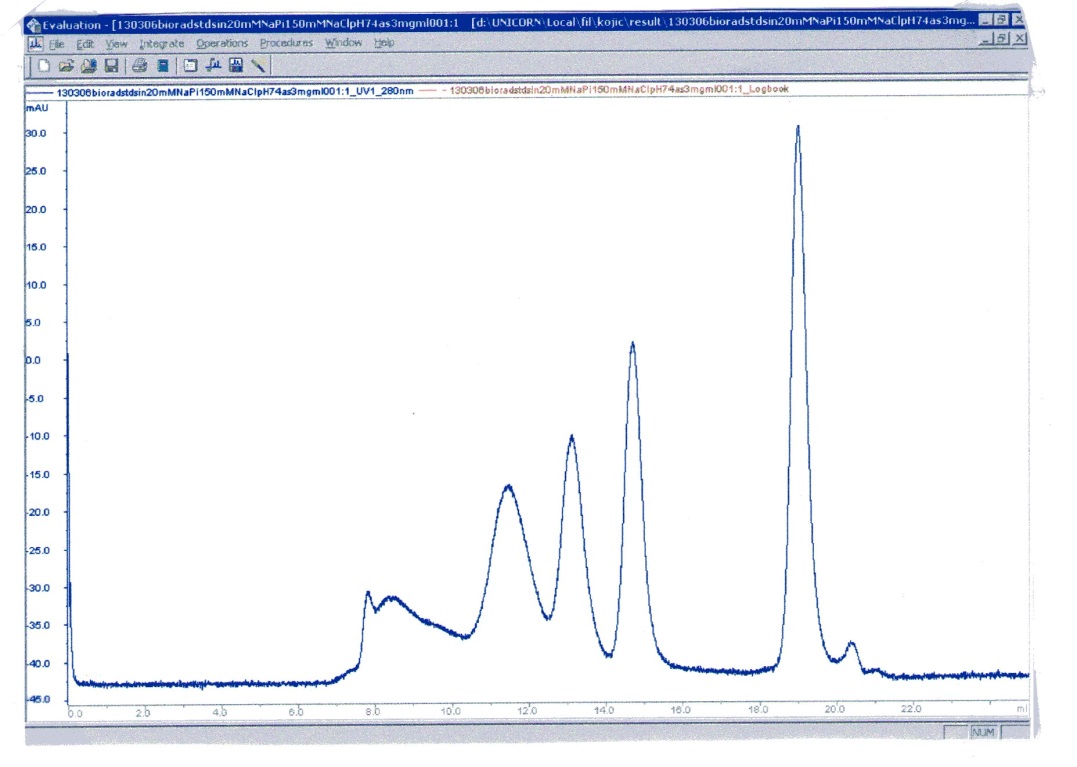


**(A)**

11.44

13.11

14.70

18.99

Elution volume (ml)

20

15

10

5

0

Absorbance 280 nm

**(B)**

**Figure S3. Analytical gel filtration calibration curve. (A)** Protein markers (Biorad) consisting of thyroglobulin (670 kDa), bovine gamma-globulin (158 kDa), chicken ovalbumin (44 kDa), equine myoglobin (17 kDa) and vitamin B_12_ (1.35 kDa) were run through the Superose-12 column. The order of the markers description follows the order of elution from left to right. The elution volumes of the protein markers, shown in ml next to the corresponding peaks, were calculated with the Unicorn 3.0 software package (Amersham Biosciences). Absorbance measured at 280 nm. Elution volumes related to the MWs (logarithmic scale) of the markers and the formula that correlates them are shown in (**B)**. Diamond-shaped points in **(B)** represent the elution volumes derived in **(A)**.
