# Supplementary material for: Structural and Functional Characterization of the Recombinant Death Domain from Death-Associated Protein Kinase
Source: PLoS One. 2013 Jul 29;8(7):e70095. doi: 10.1371/journal.pone.0070095 (PMC3726526; doi:10.1371/journal.pone.0070095)
Supplement: Figure S4 — CD spectra of DAPk-DD at 5, 25 and 95°C. CD spectra of GB1-corrected DAPk-DD(L) at 5°C (blue), 25°C (green) and 95°C (red). The CD spectra show no significant profile differences or signs of protein aggregation. A noticeable reduction in MRE values at the highest temperature is caused by the thermal denaturation of the secondary structure of the DAPk-DD. Error bars are estimates of uncertainties derived from the three scans recorded per sample. (DOCX) [file pone.0070095.s004.docx]

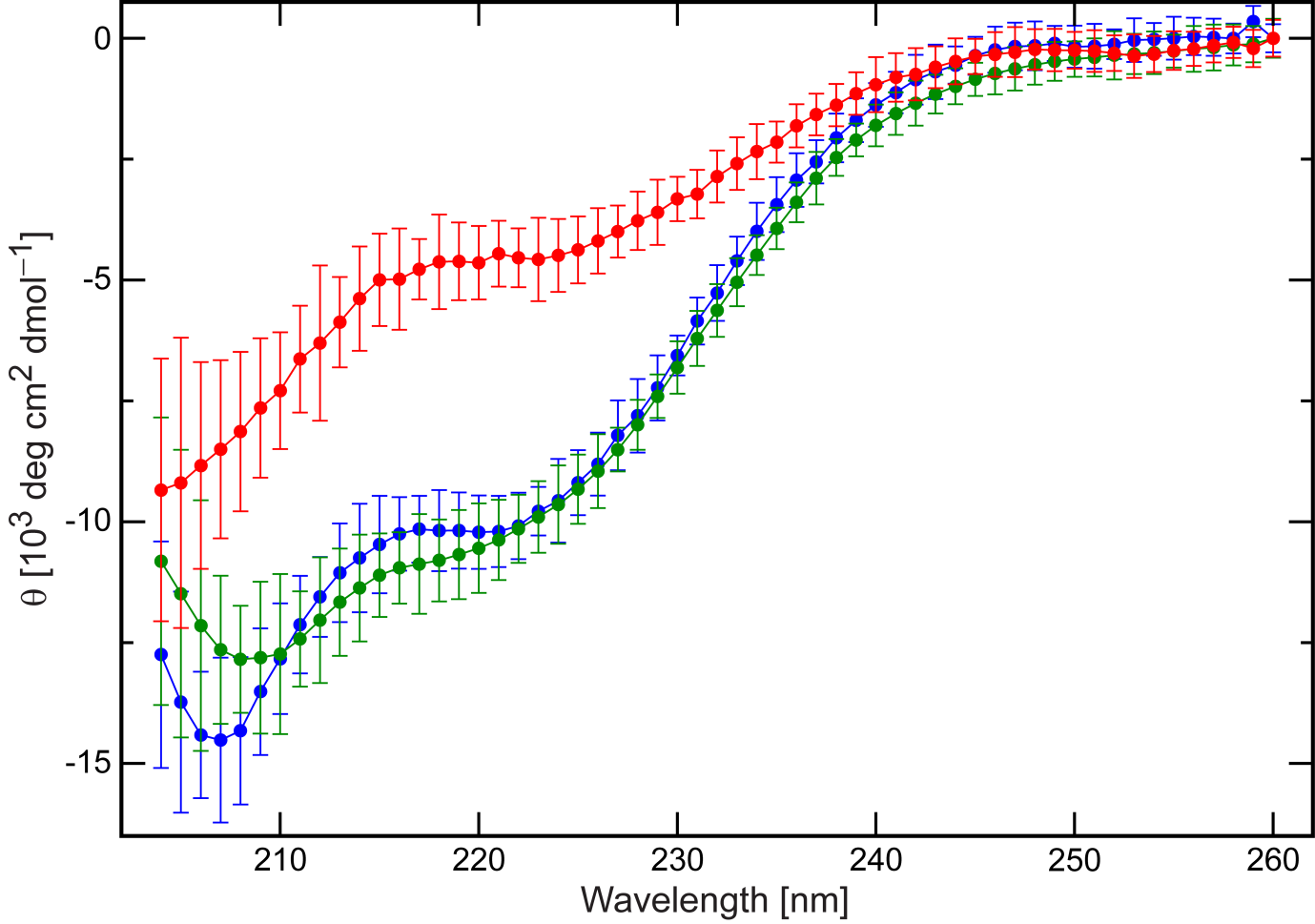


**Figure S4. CD spectra of DAPk-DD at 5, 25 and 95 °C.** CD spectra of GB1-corrected DAPk-DD(L) at 5 °C (blue), 25 °C (green) and 95 °C (red). The CD spectra show no significant profile differences or signs of protein aggregation. A noticeable reduction in MRE values at the highest temperature is caused by the thermal denaturation of the secondary structure of the DAPk-DD. Error bars are estimates of uncertainties derived from the three scans recorded per sample.
