# Supplementary material for: Structural and Functional Characterization of the Recombinant Death Domain from Death-Associated Protein Kinase
Source: PLoS One. 2013 Jul 29;8(7):e70095. doi: 10.1371/journal.pone.0070095 (PMC3726526; doi:10.1371/journal.pone.0070095)
Supplement: Figure S5 — GB1-DAPk-DD shows little secondary structure change under different buffer conditions. Far-UV CD data of the GB1-subtracted native DAPk-DD(L) in different buffers. The effect of extreme pH (red symbols = pH 2; green symbols = pH 7.4; blue symbols = pH 12) on the secondary structure of the DAPk-DD(L) is shown either in the presence of 150 mM NaCl (A) or in the absence of NaCl (B). Only at pH 2 in the absence of salt (B, red symbols) did the DD show substantial changes in structure. (C) At pH 7.4, the influence of the detergent SDS was examined. Only minor differences were seen in the CD spectra recorded on GB1-DAPk-DD(L) samples measured in the absence of SDS (green), 2 mM (red; below CMC) and 50 mM (blue; above CMC) SDS. The result indicates that SDS induced only slight secondary structure changes to the DD both below and above the CMC. (DOCX) [file pone.0070095.s005.docx]

**Figure S5. GB1-DAPk-DD shows little secondary structure change under different buffer conditions.** Far-UV CD data of the GB1-subtracted native DAPk-DD(L) in different buffers. The effect of extreme pH (red symbols = pH 2; green symbols = pH 7.4; blue symbols = pH 12) on the secondary structure of the DAPk-DD(L) is shown either in the presence of 150 mM NaCl (**A**) or in the absence of NaCl (**B**). Only at pH 2 in the absence of salt (B, red symbols) did the DD show substantial changes in structure. (**C**) At pH 7.4, the influence of the detergent SDS was examined. Only minor differences were seen in the CD spectra recorded on GB1-DAPk-DD(L) samples measured in the absence of SDS (green), 2 mM (red; below CMC) and 50 mM (blue; above CMC) SDS. The result indicates that SDS induced only slight secondary structure changes to the DD both below and above the CMC.
