# Supplementary material for: Structural and Functional Characterization of the Recombinant Death Domain from Death-Associated Protein Kinase
Source: PLoS One. 2013 Jul 29;8(7):e70095. doi: 10.1371/journal.pone.0070095 (PMC3726526; doi:10.1371/journal.pone.0070095)
Supplement: Figure S6 — The interaction of the DAPk-DD with ERK2 does not lead to any significant change in the secondary structure of the DAPk-DD or ERK2 proteins. CD analysis of the ERK-2-DAPk-DD complex. Far-UV CD spectra of ERK2 (blue circles) and GB1-corrected DAPk-DD(L) (green circles) are presented. The signal for the equimolar complex of GB1-DAPk-DD(L) and ERK2 was recorded (black circles), from which the ellipticity of GB1 was subtracted to give the native DAPk-DD-ERK2 CD signal. By combining the individual ERK2 and DAPK-DD CD data (red circles) a near identical CD profile was obtained to that observed for the complex, indicating negligible changes in secondary structure to either protein upon formation of a complex. The error bars are estimates of uncertainties derived from the three scans recorded per sample. The inset represents the data over the wavelength range of 200−230 nm. (DOCX) [file pone.0070095.s006.docx]

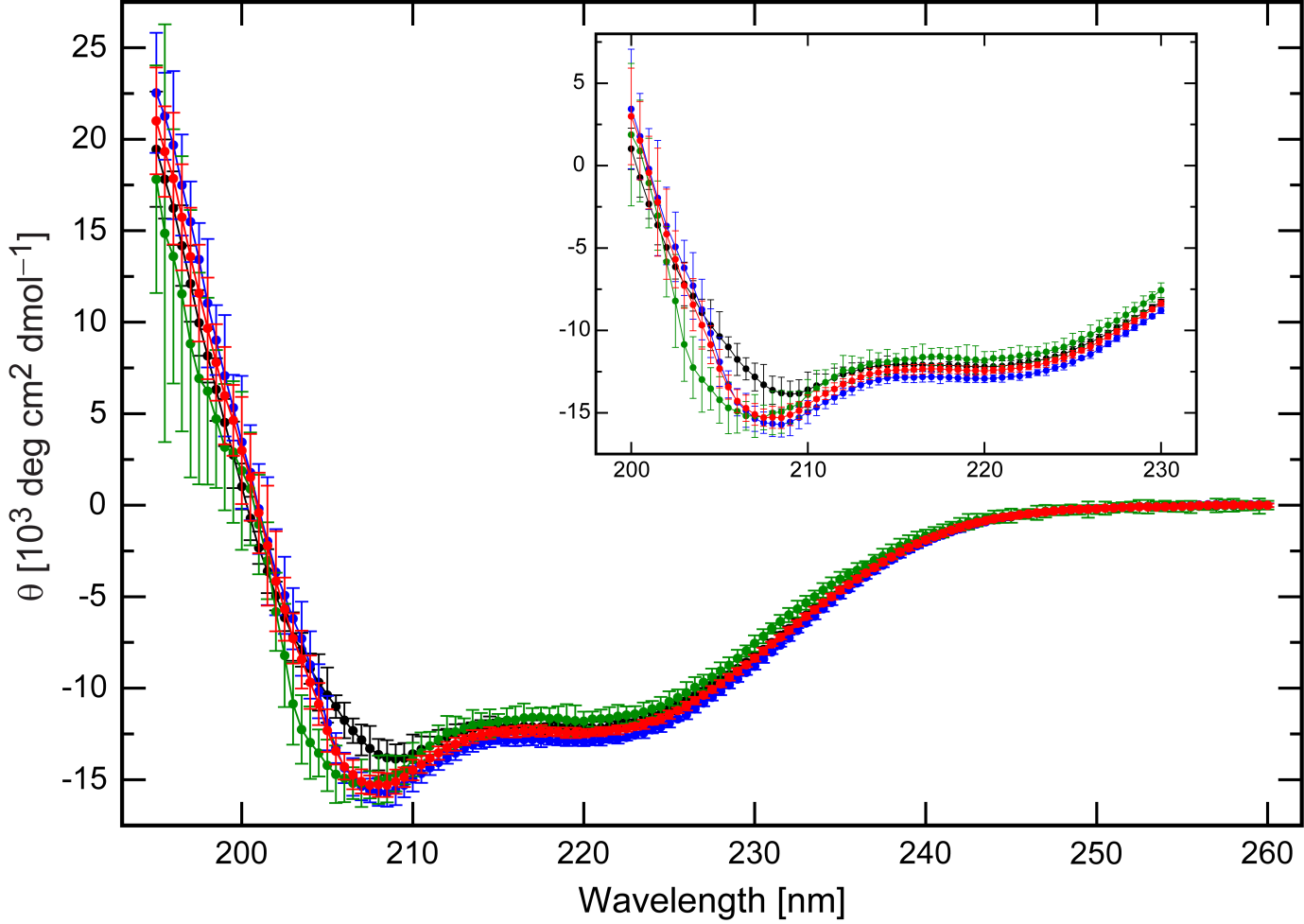


**Figure S6. The interaction of the DAPk-DD with ERK2 does not lead to any significant change in the secondary structure of the DAPk-DD or ERK2 proteins.** CD analysis of the ERK-2-DAPk-DD complex. Far-UV CD spectra of ERK2 (blue circles) and GB1-corrected DAPk-DD(L) (green circles) are presented. The signal for the equimolar complex of GB1-DAPk-DD(L) and ERK2 was recorded (black circles), from which the ellipticity of GB1 was subtracted to give the native DAPk-DD-ERK2 CD signal. By combining the individual ERK2 and DAPK-DD CD data (red circles) a near identical CD profile was obtained to that observed for the complex, indicating negligible changes in secondary structure to either protein upon formation of a complex. The error bars are estimates of uncertainties derived from the three scans recorded per sample. The inset represents the data over the wavelength range of 200−230 nm.
